# Dopaminergic Neuromodulation of Spike Timing Dependent Plasticity in Mature Adult Rodent and Human Cortical Neurons

**DOI:** 10.3389/fncel.2021.668980

**Published:** 2021-04-22

**Authors:** Emma Louise Louth, Rasmus Langelund Jørgensen, Anders Rosendal Korshoej, Jens Christian Hedemann Sørensen, Marco Capogna

**Affiliations:** ^1^Department of Biomedicine, Aarhus University, Aarhus, Denmark; ^2^DANDRITE, The Danish Research Institute of Translational Neuroscience, Aarhus University, Aarhus, Denmark; ^3^Department of Neurosurgery, Aarhus University Hospital, Aarhus, Denmark; ^4^Center for Proteins in Memory–PROMEMO, Danish National Research Foundation, Aarhus University, Aarhus, Denmark

**Keywords:** dopamine, human cortical slices, layer 5 pyramidal neurons, spike timing dependent plasticity, synaptic inhibition

## Abstract

Synapses in the cerebral cortex constantly change and this dynamic property regulated by the action of neuromodulators such as dopamine (DA), is essential for reward learning and memory. DA modulates spike-timing-dependent plasticity (STDP), a cellular model of learning and memory, in juvenile rodent cortical neurons. However, it is unknown whether this neuromodulation also occurs at excitatory synapses of cortical neurons in mature adult mice or in humans. Cortical layer V pyramidal neurons were recorded with whole cell patch clamp electrophysiology and an extracellular stimulating electrode was used to induce STDP. DA was either bath-applied or optogenetically released in slices from mice. Classical STDP induction protocols triggered non-hebbian excitatory synaptic depression in the mouse or no plasticity at human cortical synapses. DA reverted long term synaptic depression to baseline in mouse via dopamine 2 type receptors or elicited long term synaptic potentiation in human cortical synapses. Furthermore, when DA was applied during an STDP protocol it depressed presynaptic inhibition in the mouse but not in the human cortex. Thus, DA modulates excitatory synaptic plasticity differently in human vs. mouse cortex. The data strengthens the importance of DA in gating cognition in humans, and may inform on therapeutic interventions to recover brain function from diseases.

## Introduction

Humans and other mammalians are characterized by their ability to produce goal-directed and intelligent behaviors beyond simple stimulus–response associations. It is believed that the neuromodulator dopamine (DA) plays a key role in gating cortical operations underlying cognitive functions, such as working memory (Arnsten et al., 2012), attention (Thiele and Bellgrove, 2018), and flexible behavior (Klanker et al., 2013). Furthermore, evidence suggests that behaviorally-relevant sensory information and contextual information are gated by DA to maintain relevant information in working memory and relay choice signals (Ott and Nieder, 2019).

DA is synthesized by specific neurons in the midbrain that send their widespread projections to several brain regions including selected areas of the cerebral cortex such as prefrontal cortex (PFC) (Björklund and Dunnett, 2007). The gating elicited by DA, released by midbrain axonal varicosities and terminal endings within the cerebral cortex, is assumed to represent a key molecular substrate underlying cognitive performance including stimulus selection, working memory, rule switching and decision making (Merten and Nieder, 2012).

Many theories have been proposed to account for by DA circuit mechanisms underlying cortical-mediated executive control (Ott and Nieder, 2019). One of the most successful is the reward prediction error theory and its experimental demonstration in DA neurons (Schultz et al., 1997).

A phenomenon that could represent a landmark cellular substrate of cognitive functions is represented by the DA modulation of spike timing dependent plasticity (STDP) (Calabresi et al., 2007; Pawlak and Kerr, 2008; Shen et al., 2008). STDP is a form of synaptic plasticity triggered by repeated pairings of single presynaptic and postsynaptic spikes (Markram et al., 1997; Bi and Poo, 1998; Debanne et al., 1998; Miller et al., 2000). It depends on the order and millisecond-precision timing of spikes: multiple pre- before-post spike pairings often evoke timing-dependent long-term potentiation (t-LTP), whereas post-before-pre pairings often evoke timing-dependent long-term depression (t-LTD). STDP is a remarkable example of Hebbian plasticity (Hebb, 1949), since synaptic inputs that promote postsynaptic firing are strengthened. This type of plasticity has been extensively characterized by the work of various groups (Feldman, 2000; Bender et al., 2006; Nevian and Sakmann, 2006; Rodríguez-Moreno and Paulsen, 2008; Banerjee et al., 2009, 2014; Andrade-Talavera et al., 2016; Pérez-Rodríguez et al., 2019; Falcón-Moya et al., 2020). However, non-hebbian STDP has also been observed, that is multiple pre before-post spike pairings elicit t-LTD (Egger et al., 1999; Fino et al., 2005; Koch et al., 2013).

It is well established that DA has an important modulatory role on STDP, as it broadens the time window for detecting coincident spiking in the pre- and postsynaptic neurons and in this way boosts the induction of t-LTP in rodent neocortical neurons (Zhang et al., 2009; Pawlak et al., 2010; Xu and Yao, 2010; Edelmann and Lessmann, 2013; Ruan et al., 2014). Furthermore, DA modulates the polarity of STDP promoting t-LTP at excitatory synapses of rodent PFC (Ruan et al., 2014) and hippocampus (Zhang et al., 2009; Edelmann and Lessmann, 2011; Yang and Dani, 2014; Brzosko et al., 2015, 2017).

However, there are two main drawbacks with the DA modulation of STDP model. First, STDP is likely to represent a phenomenon occurring during development with a role in synaptic connections refinement in the hippocampus (Banerjee et al., 2014; Andrade-Talavera et al., 2016; Pérez-Rodríguez et al., 2019; Falcón-Moya et al., 2020) and neocortex (Corlew et al., 2007; Larsen et al., 2011, 2014). Therefore, studies investigating the role of DA on STDP has been usually performed in developmental and juvenile rodents (usually 2–3 weeks old) (reviewed by: Brzosko et al., 2019). Second, researchers wish to use STDP to model synaptic plasticity occurring at human cortical neurons (Müller-Dahlhaus et al., 2010), since this form of synaptic plasticity can be demonstrated at excitatory synapses of human hippocampus (Testa-Silva et al., 2010) and cortex (Verhoog et al., 2013). However, it is still unknown whether DA modulates STDP at human neocortical synapses.

We aimed to make progress on these two issues by testing the DA modulation of STDP from neurosurgically-resected adult human neocortical slices and by comparing it to the DA modulation of STDP in mature adult mouse cortical synapses.

In brief, we found a more pronounced non-hebbian t-LTD evoked by extracellular stimulation with intact synaptic inhibition in layer 5 pyramidal neurons of the neocortex from mature adult mice compared to mature adult neurological patients. The neuromodulator DA switched t-LTD into no change in the EPSP via DA type 2 receptor or evoked t-LTP after the pairing protocol in mature adult mice or human patients, respectively. We explain these differences by species-specific differential DA modulation of synaptic inhibition.

## Materials and Methods

### Animals

C57B/6J mice and DAT^IREScre^ mice were purchased from Jackson Laboratory and bred in-house in the animal facility of the Department of Biomedicine, Aarhus University. Mice were group housed in a temperature and humidity controlled plastic vivarium in a 12 h regular light/dark cycle with lights on at 8:00 a.m. All procedures with animals were approved by and conducted in accordance to with the Animal Experiments Inspectorate under the Ministry of Environment and Food of Denmark (License number 2017-15-0201-01201). This study was carried out in compliance with the ARRIVE guidelines^1^.

### Viral Transfection

Nine DAT^IREScre^ mice (P35-45) from four litters were anesthetized using a mix of 0.05 mg/ml of Fentanyl [Hameln pharma Ltd., United Kingdom; 0.05 mg/kg), plus 5 mg/ml of Midazolam (Hameln Pharma Ltd., United Kingdom) 5 mg/kg] and 1 mg/ml of medetomidine (0.5 mg/kg; VM Pharma). They then received bilateral injections of ssAAV-9/2-hEF1α-dlox-hChR2(H134R)_EYFP(rev)-dlox-WPRE-hGHp**(A)** (1–2 μL; titer: 6.0 × 10∧12 vg/ml; VVF, Switzerland) into the VTA at the following coordinates: anterior-posterior + 3.1 mm, medio-lateral ± 0.5 mm, dorsal-ventral −4.5 mm with respect to Bregma. Injections were made through a pulled 1 mm glass pipette using a Picospritzer III (Parker Hannifin, United States). Pipettes were kept in place for at least 10 min after injection of the virus. Following surgery, animals received 0.1 mg/kg of Buprenorphine (Temgesic; Indivior UK Limited) subcutaneously and an antidote mix of 0.4 mg/ml Naloxone (B. Braun, 115241) 1.2 mg/kg), plus 5 mg/ml of Atipamelozone Hydrochloride (2.5 mg/kg) and 0.5 mg/ml of Flumazenil [(Hameln Pharma Ltd., United Kingdom) 0.5 mg/kg] to reverse the anesthesia. Then, mice were single-housed. We recorded from VTA neurons 3 weeks after surgery from two animals. Furthermore, we recorded neurons in the prefrontal cortex 6 weeks after surgery from seven animals. This extra time was necessary to be able to detect the virus expression in fibers of the prefrontal cortex (Supplementary Figure 5C).

### Human Tissue Acquisition

All procedures with human tissue and data were approved by and conducted in accordance with the Scientific Ethics Committee for the Region of Midtjylland Denmark (official name in Danish: De Videnskabsetiske Komitéer for Region Midtjylland) (project number M-2017-82-17). All patients provided informed consent for tissue donation. Human brain tissue samples were obtained from Aarhus University Hospital in collaboration with the neurosurgery team. Patients in this study were undergoing surgery for a deep brain tumor and the samples we received were from surgically excised tissue that needed to be removed in order to gain access to the tumor. The sample provided was taken as far from the tumor as was feasible, this was typically 5–10 mm. Samples were taken from the temporal, parietal, occipital and frontal lobes. See Table 2 for more detailed information of the patients involved in this study.

Human brain tissue samples were surgically excised and immediately placed in ice-cold *N*-methyl-D-glucamine (NMDG)-based artificial cerebral spinal fluid (aCSF). NMDG aCSF was prepared as previously described (Ting et al., 2018), the composition in mM was 92 NMDG, 2.5 KCl, 1.25 NaH2PO4, 30 NaHCO3, 20 4-(2-hydroxyethyl)-1-piperazineethanesulfonic acid (HEPES), 25 glucose, 2 thiourea, 5 Na-ascorbate, 3 Na-pyruvate, 0.5 CaCl_2_⋅4H_2_O and 10 MgSO4⋅7H_2_O. The pH was titrated to 7.3–7.4 with hydrochloric acid and the osmolality was 300–310 mOsmoles/Kg. The solution was chilled on ice and bubbled with carbogen gas. Once the tissue was taken out of the operating room, we removed any excess blood and white matter before placing the sample into a fresh tube of oxygenated NMDG aCSF. The sample was placed on ice, connected to a portable container of carbogen gas and transported to the laboratory (∼15 min travel time).

### Acute *ex vivo* Brain Slice Preparation

Mice were killed by decapitation while under isoflourane anesthesia. The brain was removed and placed in ice-cold NMDG based aCSF for approximately 2 min. Mouse brains were blocked and mounted on the vibrating microtome platform to cut coronal sections of the temporal cortex, parietal cortex, prefrontal cortex or VTA. Human brain samples were mounted such that the blade was normal to the pial surface. We did not remove the pia mater. From this point, the procedure for preparation of adult mouse and human acute *ex vivo* brain slices was the same. Acute brain slices 350 μm in thickness were sliced on a Leica 1200S vibrating mictrome (Leica Microsystems, Denmark). Slices were then placed in a recovery chamber containing 32°C, carbogenated NMDG aCSF for 12 min. Slices were then transferred to a holding chamber with room temperature carbogenated aCSF composed (in mM) of: 92 mM NaCl, 2.5 mM KCl, 1.25 mM NaH2PO4, 30 mM NaHCO3, 20 mM HEPES, 25 mM glucose, 2 mM thiourea, 5 mM Na-ascorbate, 3 mM Na-pyruvate, 2 mM CaCl2⋅4H2O, and 2 mM MgSO4⋅7H2O, with a pH of 7.3, and osmolality of 300–310 mOsmoles/Kg. Slices were left to recover for at least 1 h before recording and were stored for up to 12 h.

### Electrophysiology

Brain slices were transferred to a recording chamber mounted on a SliceScope microscope (Scientfica, United Kingdom) and superfused with carbogenated ACSF composed of 119 mM NaCl, 2.5 mM KCl, 1.25 mM NaH_2_PO_4_, 24 mM NaHCO3, 12.5 mM glucose, 2 mM CaCl_2_⋅4H_2_O, and 2 mM MgSO_4_⋅7H_2_O. Pyramidal cells in layer V of the mouse or human temporal, parietal or prefrontal cortex were visualized with infrared differential interference contrast microscopy. In total, recordings from wild type mice used for STDP protocol were obtained from 56 prefrontal, 21 temporal and 21 parietal cortex. Recordings from human cortical neurons were obtained from 22 temporal, 1 occipital, 4 parietal and 2 frontal cortex. In the majority of the experiments, we cut slices from human tissue with similar orientation and similar size. No effort was made to remove the pia and cortical slices were cut with the pia perpendicular to the blade. A SciCam Pro camera (Scientifica, United Kingdom) was used for visualization and image capture. Whole-cell recordings were performed using borosilicate glass pipettes pulled with a horizontal pipette puller (DMZ universal electrode puller, Zeitz, Germany). Pipettes contained intracellular solution consisting of 126 mM K-gluconate, 10 mM HEPES, 4 mM KCl, 4 mM Mg-ATP, 0.3 mM Na3-GTP and 10 mM Na2-phosphocreatine. Liquid junction potential was calculated to be −16 mV and was not corrected for. For recordings of IPSCs only, intracellular solution consisted of 65 mM CsMeSO3, 65 mM K-gluconate, 10 mM HEPES, 10 mM CsCl2, 4 mM MgCl, 0.1 mM EGTA, 2 mM Mg-ATP, 0.3 mM Na3-GTP, 10 mM Na2-phosphocreatine. Liquid junction potential was calculated to be −11 mV and was not corrected. For both solutions the pH was 7.3–7.4 and 290 mOsmoles/Kg. When the electrodes were filled with an internal solution had an estimated resistance ranging 3–5 MΩ. Recordings were made using a Multiclamp 700B amplifier, acquired at 20 kHz, low-pass filtered at 2 kHz using a Digidata 1550B low noise data acquisition system (Molecular Devices, United States). For whole-cell recordings, pipette capacitance was neutralized and bridge balance applied. Recorded cells had an initial resting membrane potential between −60 and −75 mV. Recordings were included only if they had a change in series resistance of < 25%. A baseline recording was obtained for at least 10 min. All experiments were performed in the absence of GABAergic transmission blockers.

### Spike Timing Dependent Plasticity Protocol

A stimulating electrode inserted in a glass pipette filled with recording aCSF with a resistance of approximately 1 MΩ was placed approximately 150 μm from the soma nearby the apical dendrite. As described in Verhoog et al. (2013), EPSPs (blocked by 2 mM kynurenic acid—Supplementary Figures 4F, 8F) with an amplitude between 3 and 8 mV or IPSCs (blocked by 1 μM gabazine—Supplementary Figure 9B) with an amplitude between 100 and 300 pA were evoked at a rate of 0.14 Hz (stimulation parameters were 100 μs duration and 200–500 μA intensity) controlled by an A360 stimulus isolator (World Precision Instruments, United Kingdom). Cells were held between −68 and −72 mV for evoked EPSPs and 0 mV for IPSCs.

Pairing was always conducted in current clamp mode, for both EPSPs and IPSPs. Various pairing protocol timings were used, from 30 ms pre- before post- synaptic stimulation to 30 ms post- before pre- synaptic stimulation. For all timings, an induction protocol of 75 pairings of the EPSP with an AP generated by direct stimulation to the cell body (5 ms duration, intensity between 500 and 1,500 pA) a rate of 0.14 Hz was used. For the burst pairing protocol, a 25 ms depolarization with an intensity between 500 and 100 pA was used to induce 3–4 APs. Dopamine (20 μM, Tocris) was bath applied for 10 min starting during the last minute of baseline recordings and lasting throughout the induction protocol. The D1R antagonist 10 μM SCH23390 (Tocris) or theD2R antagonist 50 μM sulpiride (Tocris) were continuously bath applied 2 min before and after the application of 20 μM DA induction protocol.

Following the induction protocol, EPSP/IPSCs were evoked at a rate of 0.14 Hz, as they were during the baseline recording. They were then monitored for the next 40–50 min for analysis. The EPSP rising slope (20–80%) and peak amplitude used for analysis were calculated from a 5 min most stable section (defined as section that best matched baseline RMP and series resistance values) between 25 and 35 min after the induction protocol. Therefore, t-LTP/t-LTD was measured with small temporal variations amongst cells within 25–35 min after the STDP protocols.

### Pharmacology

To confirm that evoked IPSCs were, in fact, GABAergic, 1 μM gabazine (SR 95531, Tocris) was applied at the end of STDP recordings. To see the effect of GABAergic synaptic transmission on EPSP kinetics, 1 μM gabazine was applied for 5 min to a separate set of mouse neurons while evoking EPSPs at a rate of 0.14 Hz, as described above. To confirm that evoked EPSCs were, in fact, glutamatergic, 2 mM kynurenic acid (Tocris) was applied at the end of STDP recordings (Supplementary Figure 8F).

To see the effect of DA bath application on EPSP kinetics and basic cellular properties, 20 μM DA was prepared in a light protected beaker to prevent oxidation by light. Dopamine was then applied for 5 min to mouse and human neurons while evoking EPSPs at a rate of 0.14 Hz, as described above.

### Optogenetics

For DAT^IREScre^ mice, endogenous release of DA during the induction protocol was achieved by stimulating ChR2 in the fibers of VTA projections. These fibers were located by eYFP expression. Terminals were stimulated with blue light from a CoolLed PE-300ultra (Scientifica) (460 nm, ∼10 mW power). Each stimulation consisted of a 1 s, 17 Hz train of blue light pulses with a 5 ms pulse width, 10 ms before the pairing of the EPSP and AP during the induction protocol.

To confirm endogenous DA release in the prefrontal cortex, we assessed the effect of blue light stimulation of ChR2 on the layer V pyramidal neuron AHP as described in Buchta et al. (2017). In brief, 5 APs were generated with a 60 ms pulse of 1,000–1,500 pA and AHP area was analyzed before and after 7 minutes of repeated blue light exposure, 1 s of 17 Hz train at 0.14 Hz to mimic DA release caused during the induction protocol. All these protocols of optogenetic stimulations were adopted from Buchta et al. (2017), who also investigated the action of DA released from VTA terminals in rodent PFC. We also stimulated VTA neurons, identified by eYFP expression, using a 10 Hz pulse train (10 ms pulse width) of blue light (Supplementary Figures 5A,B) to confirm expression of ChR2.

### Quantification of Dopamine Receptor Expression

Using publicly available data from the Allen Institute for Brain Science, we extrapolated the single nucleus mRNA expression data for DA receptor 1 and 2 (DRD1 and DRD2, respectively). The database used can be found at https://portal.brain-map.org/atlases-and-data/rnaseq and detailed methods of single nuclei fluorescence-activated cells sorting (FACS) isolation followed by Smart-seq v4 based library preparation and single-cell deep (2.5 million reads/cell). RNA-Seq can be found in Hodge et al. (2019). Data was derived from human medial temporal gyrus tissue samples and adult mouse primary visual cortex and anterior lateral motor area (postnatatal days 53–59). The data was organized by cortical layer expression in GABAergic neurons. Plots were made using GraphPad prism and represent the distribution of mRNA expression on a log scale of counts per million (CPM).

### Data Analysis

All electrophysiological data was acquired using pCLAMP 10.7 and analyzed in clampfit 10.7 (Molecular Devices, United States). Statistical analysis was performed using GraphPad Prism 8 (GraphPad Software, United States). All EPSP/IPSC rising slopes and amplitudes, and basic electrophysiological properties were normalized to baseline for analysis. All data are presented as mean ± SEM. Due to the nature of whole-cell electrophysiology and availability of human tissue, there is a limited sample size (number of recorded neurons). When comparing two groups, the Mann Whitney *U*-test was used. When comparing the effect of STDP to baseline a one sample Wilcoxon signed rank test was used. When more than two groups were compared, a Kruskal-Wallis Test was used. No more than two neurons using the same protocol were sampled from the same mouse or human brain sample.

## Results

### Excitatory Synapses Show t-LTD in Mature Adult Mice

**FIGURE 1:**
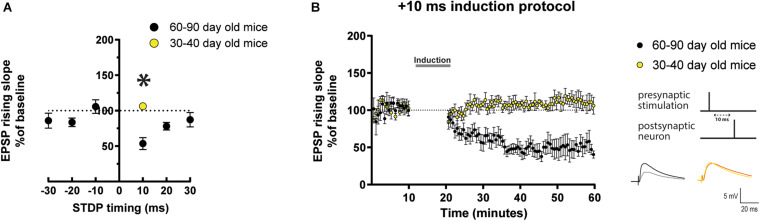
STDP induction at varying timings in layer 5 cortical pyramidal neurons of mature adult mice and comparison to adult mice. **(A)** STDP induction at EPSP-AP pairing timings (Δτ) of –30 ms, –10 ms, and + 30 ms show no change in EPSP rising slope. At Δτ = + 10 ms neurons from mature adult mice (60–80 days old) exhibit t-LTD (*p = 0.008 vs. 100%) which was significantly different from adult mice (30–40 days old) (*p* = 0.0007). Neurons from adult mice exhibited no change (*p* = 0.094 vs. 100%). **(B)** The graph shows the time-course of the EPSP rising slope during the STDP experiments performed in both mature adult and adult mice. The gray bar in the graph illustrates the time of STDP induction. To the right, the STDP induction protocol timing is illustrated and below are example traces of EPSPs. The EPSPs were evoked at a rate of 0.14 Hz throughout the protocol. The baseline trace is the darker trace, the traces following STDP induction are the lighter traces; each trace is the average of 80 traces from the same recording. All data are shown as mean ± SEM. Number of neurons recorded in mature adult mice, *n* = 8 (4 prefrontal, 2 parietal and 2 temporal cortex), and in adult mice *n* = 6 (4 prefrontal, 1 parietal and 1 temporal cortex).

In order to characterize STDP in mature adult mice, we tested different STDP timings (Δτ = −30 ms, −20 ms, −10 ms, + 10 ms, + 20 ms, and + 30 ms, where a + Δτ is pre before post-synaptic stimulation) in 60–90 day old mice (Figure 1A). We patched layer V pyramidal neurons in whole-cell configuration and placed a stimulating electrode approximately 100–150 μm from the soma, nearby the apical dendrite, without pharmacological blockade of synaptic inhibition to mimic physiological conditions. We first recorded a 10 min baseline of evoked excitatory postsynaptic potentials (EPSPs), followed by an STDP induction protocol consisting of 75 EPSPs to action potential (AP) pairings. We then monitored evoked EPSPs for another 40 min (Figure 1B). For the baseline, induction and further monitoring, EPSPs were evoked at a rate of 0.14 Hz. We did not observe significant effects induced by Δτ 30 ms (Figure 1A, −30 ms: 85.9 ± 10.4% vs. 100%, *n* = 6, *p* = 0.4; + 30 ms: 87.2 ± 10.1% vs. 100%, *n* = 6, *p* = 0.3. We also observed no change at Δτ = −20 ms (Figure 1A, 83.5 ± 6.1% vs. 100%, *n* = 6, *p* = 0.06) and Δτ = −10 ms (Figure 1A, 105.8 ± 9.5% vs. 100%, *n* = 6, *p* = 0.6). In contrast, at Δτ = + 10 ms neurons exhibited t-LTD where EPSPs had a decreased rising slope (Figures 1A,B, 53.6 ± 8.4% vs. 100%, *n* = 8, *p* = 0.008) and peak amplitude (Supplementary Figure 1). At Δτ = + 20 ms (Figure 1A, 7.1 ± 5.3% vs. 100%, *n* = 6, *p* = 0.03) we observed t-LTD. Interestingly, we found that at mature adult excitatory synapses, the plasticity outcome was dependent on the strength of postsynaptic depolarization, as reported for younger mice (Meredith et al., 2003). Experimentally, we replaced a single spike Δτ = + 10 ms induction with a 25 ms depolarization protocol to produce 3–4 APs as shown in Supplementary Figure 2. We found that such burst protocol instead of producing t-LTD, as observed with a postsynaptic single spike protocol, resulted in no change in EPSP rising slope or peak amplitude. We also tested a 20 Hz stimulation protocol and found that this also produced no change in EPSP rising slope (Supplementary Figure 2B).

Next, we compared the result observed after the Δτ = + 10 ms STDP protocol in mature adult mice (60–90 day old) and young adult mice (30–40 day old). We found that there was a significant difference in the result observed with Δτ = + 10 ms STDP induction in these two groups of mice as seen in the EPSPs rising slope (Figures 1A,B, mature adult: 53.6 ± 8.4%, *p* = 0.008, *n* = 8; adult: 106.3 ± 2.6% vs. 100%, *p* = 0.094, *n* = 6) and peak amplitude (Supplementary Figure 1). It is important to notice that this result detected in adult mice (30–40 day old) reproduces the previously reported lack of lasting changes of EPSPs observed in 30–50 day old mice in similar experimental conditions, i.e., EPSPs evoked in layer 5 pyramidal neurons with intact synaptic inhibition (Xu and Yao, 2010).

### Dopamine Application Blocks t-LTD in Mature Adult Mice

**FIGURE 2:**
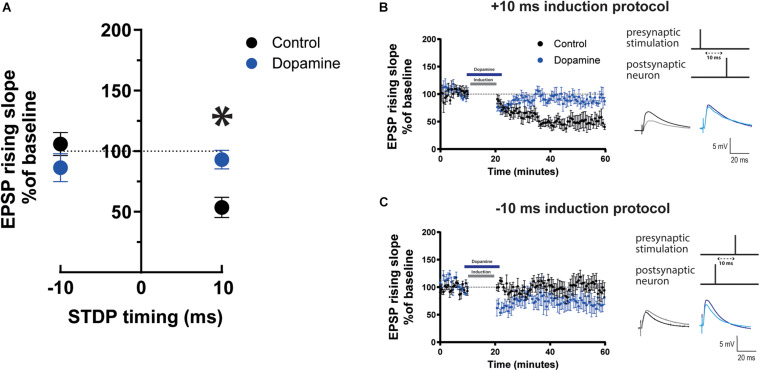
Effect of dopamine on EPSP rising slope following STDP induction in mature adult mouse cortical layer 5 pyramidal neurons. **(A)** STDP induction at Δτ = –10 ms and + 10 ms EPSP-AP pairing timings with and without 20 μM dopamine application. EPSP rising slope with the –10 ms timing was not different between the control and dopamine group (*p* = 0.4). At the + 10 ms timing, EPSP rising slope showed a significant change (*p = 0.01) from t-LTD to no change. The time-course of the EPSP rising slope during the STDP experiment using the + 10 ms timing is shown in **(B)** and the –10 ms timing in **(C)**. Dopamine bath application and the time of STDP induction are indicated by bars in the graphs. To the right, the STDP induction protocol timing is illustrated and below are example traces of EPSPs. The EPSPs were evoked at a rate of 0.14 Hz throughout the protocol. The baseline trace is the darker trace, the resultant trace following STDP induction is the lighter trace; each trace is the average of 80 traces from the same recording. All data are shown as mean ± SEM. For the –10 ms timing *n* = 6 and for the + 10 timing *n* = 8. Overall, data are from *n = 28*, 20 prefrontal, 4 parietal and 4 temporal recorded cells (Figure 2A); *n = 16*, 8 prefrontal, 4 parietal and 4 temporal recorded cells (Figure 2B); *n = 12*, all prefrontal recorded cells (Figure 2C).

After determining that STDP induction has an age dependent effect on EPSPs, we sought to investigate whether bath application of a cortical neuromodulator such as DA would modulate the STDP effect toward t-LTP in mature adult mice (60–90 day old) as previously shown in younger mice (Xu and Yao, 2010; Brzosko et al., 2015). DA (20 μM) was applied for 10 min starting during the last minute of baseline recordings and lasting throughout the induction protocol. We found that the EPSPs recorded from cells where DA was applied during the Δτ = + 10 ms STDP induction protocol had a significantly steeper rising slope (Figures 2A,B) and amplitude (Supplementary Figures 3A,B) than those recorded from cells that underwent the induction protocol in the absence of DA. As a result, DA changed the t-LTD seen in mature adult mice to no change in EPSP rising slope (Figures 2A,B, 93.02 ± 7.7, *p* = 0.5 vs. 100%, *n* = 8) and peak amplitude (Supplementary Figures 3A,B). This modulatory effect by DA was abolished in the presence of the DA receptor type 2 (D2R) antagonist sulpiride (50 μM), but was not affected by the DA receptor type 1 (D1R) antagonist SCH23390 (10 μM) (Supplementary Figure 13). This pharmacological result suggests that the DA modulation of STDP act through D2Rs.

In contrast, when DA was applied during the Δτ = −10 ms STDP induction protocol, EPSP rising slope (Figures 2A,C, 86.4 ± 11.5 vs. 100%, *p* = 0.4, *n* = 6) and peak amplitude (Supplementary Figures 3A,C) remained unchanged and there was no difference in EPSP rising slope (Figures 2A,C), or peak amplitude (Supplementary Figures 3A,C) before and after DA. Furthermore, DA (20 μM) application did not affect the rising slope of EPSPs evoked at 0.14 Hz with no STDP induction protocol (Supplementary Figure 4), and did not significantly affect AP firing frequency and basic electrophysiological properties (Supplementary Figure 4).

The results show that DA modulates STDP by blocking t-LTD in mature adult mice.

### Optogenetically Triggered Release of Dopamine Also Blocks t-LTD

**FIGURE 3:**
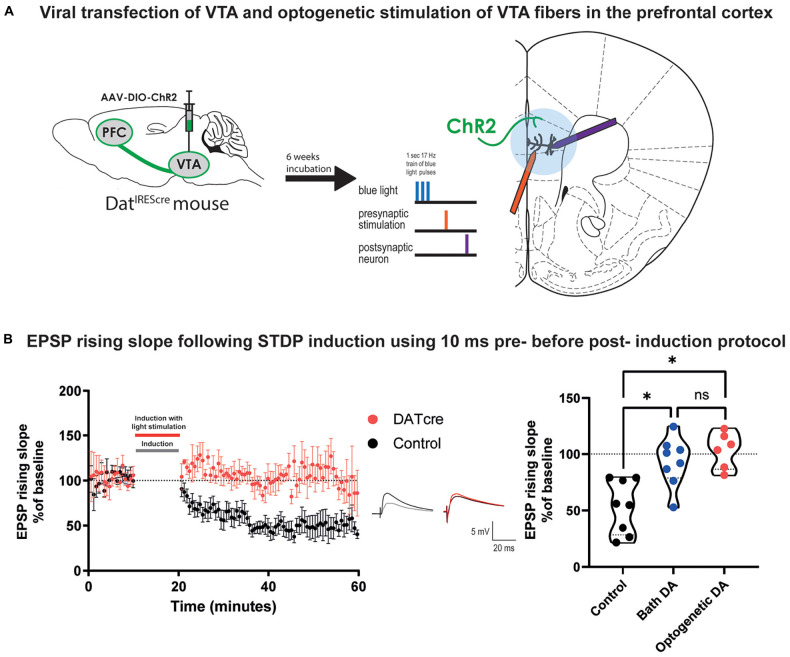
Optogenetically triggered DA release during STDP induction blocks EPSP rising time t-LTD in cortical layer 5 pyramidal neurons of mature adult mouse. **(A)** Scheme of the viral transfection (left) and the electrophysiological protocol for STDP induction (right). Briefly, ChR2 expressing fibers from dopaminergic neuron in the VTA (shown in green) were stimulated using blue light during the STDP induction protocol. **(B)** The time-course of the EPSP rising slope during the STDP experiment in both control and Dat^IREScre^ mice (left) and violin plots of the summary of the data, (right). Data demonstrate that optogenetically triggered release of DA (blue light pulses, 460 nm, ∼10 mW power) had a similar effect to bath applied DA (Dunn’s multiple comparison test, *p* = 0.99) where there is a significant difference between the control group and the DA exposed groups (Dunn’s multiple comparison test, control vs. bath application of DA: *p = 0.04, control vs. optogenetic DA: *p = 0.004). The EPSPs were evoked at a rate of 0.14 Hz throughout the protocol. In the middle example traces of EPSPs are shown. The baseline trace is the darker trace, the resultant trace following STDP induction is the lighter trace; each trace is the average of 80 traces from the same recording. All data are shown as mean ± SEM. For the optogenetic stimulation *n* = 6, while for the control and bath DA groups *n* = 8.

Bath application of drugs can be not entirely representative of physiological conditions. In order to test the effect by DA using more physiologically relevant conditions, we expressed channelrhodopsin (ChR2) in the ventral tegmental area (VTA) dopaminergic neurons using Dat^IREScre^ mice (Figure 3A). Expression of ChR2 was confirmed by patching fluorescently identified VTA cells and stimulating with blue light pulses (460 nm, ∼10 mW power) as in Supplementary Figures 5A,B. After 6 weeks of transfection, fibers from VTA neurons were visible in the prefrontal cortex (Supplementary Figure 5C). We determined that EPSP rising slope in layer V pyramidal neurons in the prefrontal cortex was not affected by blue light stimulation alone (Supplementary Figure 5D). To confirm DA release from fibers we analyzed afterhyperpolarization (AHP) area occurring after 5 APs evoked by a depolarizing current pulse before and after blue light stimulation. We found that the AHP area was decreased following optogenetically triggered DA release (Supplementary Figures 5E,F,G), as previously reported (Buchta et al., 2017).

We then performed STDP experiments using the Δτ = + 10 ms induction protocol on layer V pyramidal neurons in the prefrontal cortex using slices from the adult Dat^IREScre^ mice. Instead of bath application of DA, five blue light pulses were added 10 ms before the EPSP-AP pairing during the induction protocol (Figure 3A). We found that similar to bath application of DA, optogenetic DA stimulation during STDP induction also prevented the induction of t-LTD of EPSP rising slope (Figure 3B, 103.3 ± 6.5, vs. 100%, *n* = 6, *p* = 0.6) and peak amplitude (Supplementary Figure 6). These results were significantly different from the t-LTD of the EPSP rising slope (Figure 3B, Kruskal-Wallis test, *p* = 0.0009) and peak amplitude (Supplementary Figure 6) observed in the control group. This result suggests that bath application of DA to prefrontal cortical slices and release of DA from VTA fibers within prefrontal cortical slices evoke similar modulation on STDP of excitatory synapses in the mature adult mouse.

### Dopamine Elicited t-LTP of Excitatory Synapses in Human Cortical Pyramidal Neurons

**FIGURE 4:**
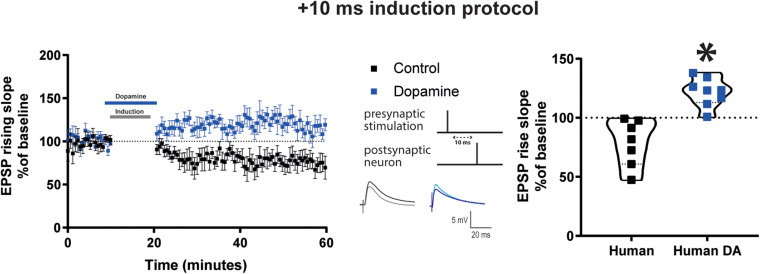
DA potentiates baseline EPSP rising time after Δτ = + 10 ms STDP protocol in adult human cortical layer 5 pyramidal neurons. Left, the time-course of the EPSP rising slope after Δτ = + 10 ms STDP induction protocol. Middle, the STDP induction protocol timing is illustrated and below are example traces of EPSPs. The EPSPs were evoked at a rate of 0.14 Hz throughout the protocol. The baseline trace is the darker trace, the resultant trace following STDP induction is the lighter trace; each trace is the average of 80 traces from the same recording. Right, violin plots of summary of the results showing a significant difference between EPSP rising slope with and without DA application during STDP induction (*p = 0.0003). All data are shown as mean ± SEM. For the control group *n* = 7 and for the DA group *n* = 8. Overall, data are obtained from 15 neurons of human neocortex, 10 temporal, 4 parietal, 1 occipital in control and DA.

Next we recorded from neurons of cortical tissue resected from mature adult patients that underwent neurosurgery to test whether observations obtained in mature adult mice could be extended to humans. The patients included in the present study were on average 64.7 ± 2.39 years old (Table 1). We found that the Δτ = + 10 ms STDP induction protocol elicited t-LTD for rising slope (Figure 4, control data, 78.7 ± 7.4, vs. 100%, *p* = 0.02, n = 7) and peak amplitude (Supplementary Figure 7, control data) of EPSPs recorded from layer 5 pyramidal neurons. Furthermore, DA bath application (20 μM) during Δτ = + 10 ms STDP induction protocol, caused t-LTP as measured by a significant increase of the EPSP rising slope (Figure 4, DA data, 121.8 ± 4.2, vs. 100%, *n* = 8, *p* = 0.008) and peak amplitude (Supplementary Figure 7, DA data).

**TABLE 1:** Basic electrophysiological properties of recorded neurons.

	Human	C57B/6 mice	Dat^IREScre^ mice
**Number**	18	59	9
**Age (human in years, mouse in days)**	65.3 ± 2.36	79.56 ± 1.78	72.78 ± 2.77
**RMP (mV)**	−68.17 ± 0.76	−67.68 ± 0.63	−68.91 ± 1.62
**Input resistance (MΩ)**	135.7 ± 15.38	131.8 ± 5.58	146.0 ± 14.59
**Capacitance (pF)**	209.1 ± 19.8	174.7 ± 8.10	159.0 ± 21.49
**Sag Ratio**	1.13 ± 0.01	1.10 ± 0.02	1.09 ± 0.03
**Spike Amplitude (mV)**	105.4 ± 1.70	107.8 ± 1.21	108.5 ± 2.01

*All data is displayed as mean ± SEM.*

Control experiments indicated that in the absence of STDP induction, DA application (20 μM) did not affect the rising slope of EPSPs evoked at 0.14 Hz, AP firing frequency and basic electrophysiological properties (Supplementary Figure 8).

### Synaptic Inhibition Contributes Toward Different Dopamine Modulation of STDP at Excitatory Synapses of Mature Adult Mice vs. Humans

**FIGURE 5:**
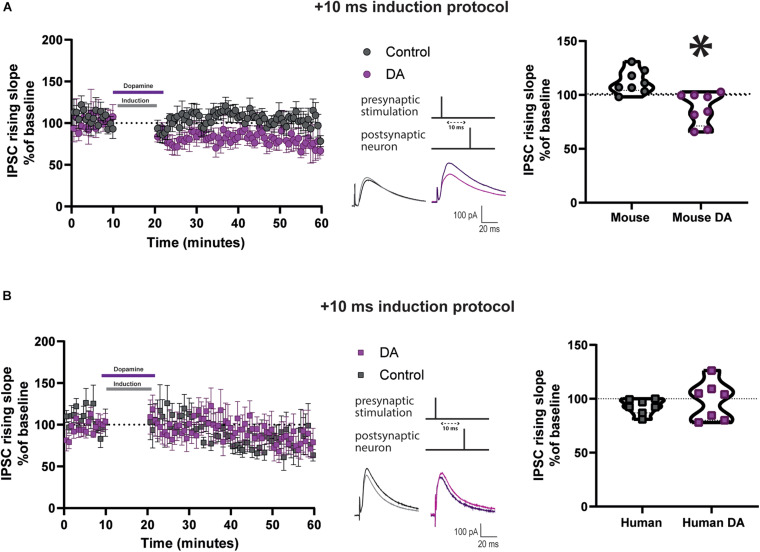
DA reduces IPSC rising slope after Δτ = + 10 ms STDP protocol in cortical layer 5 pyramidal neurons of mature adult mice but not humans. Effect of DA on IPSC rising slope following STDP induction in adult mouse **(A)** and human **(B)** pyramidal neurons. Left, the time-course of the IPSC rising slope after Δτ = + 10 ms STDP timing protocol. Middle, the STDP induction protocol timing is illustrated and below are example traces of IPSCs. The IPSCs were evoked at a rate of 0.14 Hz throughout the protocol. The baseline trace is the darker trace, the resultant trace following STDP induction is the lighter trace; each trace is the average of 80 traces from the same recording. Right, violin plots showing summary of the results. DA significantly reduced the IPSC rising slope after STDP induction in neurons recorded from mature adult mice (*p = 0.001, *n* = 8), but not from humans (*p* = 0.6, *n* = 7). All data are shown as mean ± SEM. Overall, data are obtained from 16 neurons of mouse neocortex, 8 prefrontal, 4 temporal and 4 parietal, and from 14 neurons of human neocortex, 12 temporal and 2 frontal.

The DA-mediated modulation of t-LTP at mature adult human but not at mature adult mouse excitatory synapses. What factors could contribute to this difference? DA receptors on mouse interneurons have been implicated in the mechanism of DA potentiation of STDP in younger mice (Xu and Yao, 2010). We first sought to determine if this was also true for excitatory synapses at mature adult mouse neurons. Before performing this test, we assessed whether synaptic inhibition was recruited by the stimulation protocol used and could impact EPSP kinetics. Application of the GABA-A receptor antagonist 1 μM gabazine increased EPSP peak amplitude and area, and did not change EPSP rising slope (Supplementary Figure 9A) recorded in mature adult mouse neurons. Then, inhibitory postsynaptic currents (IPSCs) were recorded in voltage clamp at 0 mV, at equilibrium potential for EPSC (Supplementary Figure 9C), before and after a STDP induction protocol, from layer V pyramidal neurons of mature adult mice. We found that the Δτ = + 10 ms STDP induction protocol, delivered in current clamp at resting membrane potential, caused an increase in the IPSC rising slope (Figure 5A, 112.2 ± 3.8, vs. 100%, *p* = 0.02, *n* = 8) but not its peak amplitude (Supplementary Figure 10A). Furthermore, DA applied during the Δτ = + 10 ms STDP induction protocol resulted in a t-LTD for the IPSC rising slope (Figure 5A; 87.2 ± 5.2, vs. 100%, *n* = 8, *p* = 0.04) and peak amplitude (Supplementary Figure 10A). When comparing the rising slope (Figure 5A) and the peak amplitude (Supplementary Figure 10A) of the IPSCs observed after STDP induction with or without DA, they were significantly different.

Data from the Allen Institute for Brain Science RNA-seq transcriptional profiles shows that DA receptors 1 and 2 have lower expression in human GABAergic neurons of cortical layer 5 than in mouse (Supplementary Figure 11). Therefore, we also examined the effect of DA application on IPSC caused by Δτ = + 10 ms STDP induction delivered in current clamp at resting membrane potential in human pyramidal neurons. The IPSCs were recorded in voltage-clamp at 0 mV, that was the reversal potential for EPSCs (Supplementary Figure 9C). We found a non-significant trend toward t-LTD after the STDP induction protocol, as assessed by the IPSC rising slope (Figure 5B, 92.9 ± 2.7, vs. 100%, *n* = 7, *p* = 0.08) and in peak amplitude (Supplementary Figure 10B). Furthermore, we observed that there was no change in IPSC rising slope (Figure 5B, 98.2 ± 6.7, vs. 100%, *n* = 7, *p* = 0.9) and peak amplitude (Supplementary Figure 10B) in neurons exposed to the t = + 10 ms STDP induction protocol in the presence of 20 μM DA. When comparing the rising slope (Figure 5B) and the peak amplitude (Supplementary Figure 10B) of the IPSCs observed after STDP induction with or without DA, they were not significantly different.

Control experiments indicated that DA alone had no effect on the rising slope of IPSCs evoked by 0.14 Hz stimulation in the absence of the STDP induction protocol in both human and mouse neurons (Supplementary Figure 12). We also confirmed that the IPSCs recorded were entirely mediated by GABA-A receptors, as application of 1 μM gabazine abolished the evoked IPSCs, as shown in examples in human and mouse neuron recordings in Supplementary Figure 9B.

Our results suggest subtle differences between STDP of synaptic inhibition and its DA modulation at layer 5 pyramidal neurons in mature adult mice compared to mature adult human patients. These differences could represent one of the mechanisms underlying the different effects by STDP of excitatory transmission and its modulation by DA in mature adult mouse vs. adult human patients.

## Discussion

The key findings of the present study can be summarized as following. We observed a non-hebbian t-LTD triggered by one post-synaptic AP preceded by pre-synaptic spiking by 10 ms (Δτ = + 10 ms) evoked by extracellular stimulation with intact synaptic inhibition in layer 5 pyramidal neurons of the neocortex from mature adult mice *in vitro*. Exogenous application of DA or optogenetic stimulation of VTA fibers, to release endogenous DA, switched this t-LTD into no change in the EPSP after the pairing protocol, an effect mediated by D2Rs. When a burst of APs (and not only one AP) were evoked in the post-synaptic neuron during the pairing protocol, then a stable EPSP (i.e., no t-LTD) was observed, presumably by transiently boosting post-synaptic dendritic calcium via back-propagating APs. Furthermore, we also investigated STDP and DA modulation in cortical pyramidal neurons from mature adult neurological patients using the same experimental conditions used in mice. We observed that the Δτ = + 10 ms protocol elicited a t-LTD response in both mature adult mouse and human layer 5 pyramidal neurons. Application of DA strengthened the EPSP toward t-LTP. In mature adult mice, an STDP protocol enhanced synaptic inhibition but this effect was reversed to t-LTD after DA application and inadult humans resulted in t-LTP.

Using classical STDP pairing protocols, we observed t-LTD after the Δτ = + 10 ms when synaptic inhibition was left unaffected in mature adult mice (60–90 day old). In contrast, the same pairing protocol performed on juvenile excitatory synapses usually evoke t-LTP in the neocortex (Markram et al., 2011) and hippocampus (Brzosko et al., 2019) of rodents. Importantly, when we tested neocortical synapses of younger mice [range 30–40 days old same as in Xu and Yao (2010)], we observed no lasting changes in the EPSP following the Δτ = + 10 ms protocol, exactly as previously reported in similar experimental conditions (Xu and Yao, 2010). The detection of different plastic rules between young adult mice (30–40 days old) and mature adult mice (60–90 days old) can be surprising, because a month old mouse is often assumed to reach adulthood and show consistent features (Flurkey et al., 2007). However, young and mature old mice can also express important differences. For example, 30 and 70 days old mice with deletion of the NMDA receptor NR1 subunit gene in glutamatergic neurons display different social behavior (Ferri et al., 2020). Furthermore, 30 day vs. 70 day old neuroligin-3 knock in mice show significant differences in the volume of cortical white and gray matter associated with altered sociability (Kumar et al., 2014).

Our data suggest that when STDP is tested under intact GABAergicinhibition, t-LTD is the prominent form of synaptic plasticitythat occurs in mature adult rodent neocortex, in contrast to whathave been seen at juvenile synapses showing t-LTP in neocortex(Markram et al., 2011) and hippocampus (Brzosko et al., 2019). As cortical layer V neurons of adult, neurons from mature mice are under powerful constraint by local GABAergic interneurons, which may explain the lack of t-LTP induced by a mild induction STDP protocol. When we applied a burst of postsynaptic APs and not only one AP in the STDP induction protocol a stable EPSP without t-LTD was observed. A previous work showed that a similar postsynaptic burst protocol to induce STDP elicited t-LTD in layer V pyramidal cells of rat somatosensory cortex (Birtoli and Ulrich, 2004). Our result is consistent with the assumption that in older animals, synapses tend to require stronger induction protocols for t-LTP to occur (Meredith et al., 2003). Of note, Meredith et al. (2003) studied neocortical synapses of rodents (up to 45 days old), an age range comparable to the study published by Xu and Yao (2010) and Ruan et al. (2014), but younger than our mature adult mouse sample.

Our results of t-LTD in mature adult mice differs from some previous reports performed in the rat, that suggested that the capacity of rodent cortical synapses to undergo STDP t-LTD decline with age (Banerjee et al., 2009; Verhoog et al., 2013). This notion is consistent with the idea that t-LTD occurs during development and plays a role is synaptic connections refinement in the hippocampus (Banerjee et al., 2009, 2014; Andrade-Talavera et al., 2016; Pérez-Rodríguez et al., 2019; Falcón-Moya et al., 2020) and neocortex (Corlew et al., 2007; Larsen et al., 2011, 2014). The difference between our data and previous results could be explained by many factors including species: rat vs. mouse; age: mature adult vs. adult or juvenile animals; brain areas tested: neocortex vs. hippocampus, as well as barrel cortex vs. parietal, prefrontal and temporal cortex (present study); neuron types: hippocampal pyramidal neuron recordings or neocortical neurons with the soma in layer 2–4 vs. layer 5 neocortical neurons (present study). It is important to note that the functional role of LTD at adult cortical synapses is still an open question and it can include at least memory storage, loss and maintenance (Massey et al., 2008; Abraham et al., 2019).

Our results obtained in mature synapses triggered by pre before-post spike pairings formally resemble non-Hebbian STDP observed in some juvenile excitatory cortical synapses. For example, pre < 25 ms before-post spike pairings of spiny stellate neurons in layer 4 of the barrel field in young rat somatosensory cortex elicits t-LTD (Egger et al., 1999). In the majority of previous studies, however, pre-postsynaptic Hebbian STDP induces t-LTP at excitatory synapses in the neocortex and hippocampus (reviewed by Feldman, 2012). A general explanation for the presence of both Hebbian and non-Hebbian t-LTD at various cortical excitatory synapses has been given some time ago (Froemke et al., 2005; Letzkus et al., 2006; Sjöström and Häusser, 2006). According to this view, the sign of synaptic plasticity in neocortical pyramidal neurons is regulated by the spread of APs to the synapse. This creates a progressive gradient between t-LTP and t-LTD as the distance of the synaptic contacts from the soma increases. Furthermore, multiple factors can determine the occurrence of t-LTP or t-LTD, such as spike rate, spike timing and number of coincident afferents (Sjöström et al., 2001) as well as initial synaptic strengths and dendritic and axonal delays propagation (Madadi Asl et al., 2018). Furthermore, we observed a lack of STDP either after pre-before-post spike pairings as well as post-before-pre pairings with a longer time interval of ± 30 ms in mature adults mice 60–90 days old consistent with previous results obtained in young adult mice (30–50 days old) (Ruan et al., 2014).

Regarding our data obtained at excitatory synapses of neocortical human cortex, they are consistent with a study documenting STDP on pyramidal neurons of layers 2–6 performed in acute slices of human cortex resected from tumor or epilepsy patients (Verhoog et al., 2013). As in our sample, this study reported that multiple pre- before-post spike pairings (especially short intervals 5–10 ms) evoke STDP t-LTD, confirming non-Hebbian STDP rules at cortical excitatory synapses. In contrast to our data, however, this study also found that post-before-pre pairings (especially at short intervals 5–10 ms) evoke STDP t-LTP. Different experimental conditions (e.g., age and patient conditions and cortical areas studied) could account for these discrepancies. It is also worth to mention that STDP induced by a pre-postsynaptic Hebbian protocol induces only t-LTP at human hippocampal Schaffer collateral CA1 pyramidal or interneuron synapses (Testa-Silva et al., 2010). This suggests that plastic rules can be different at human hippocampal compared to neocortical excitatory synapses, an issue that future work should carefully address.

Our results show that DA promoted synaptic strengthening in both mouse and human cortical excitatory synapses, but t-LTP was observed only at human cortical synapses and not at mouse cortical synapses where synaptic responses remained close to the baseline after the Δτ = + 10 ms protocol. Our observations are well aligned with the idea that DA controls the polarity of STDP (Brzosko et al., 2019). For example, DA applied during STDP induction leads to t-LTP with spike timing that would induce t-LTD in control conditions in hippocampal CA1 pyramidal neurons (Zhang et al., 2009; Brzosko et al., 2015). This modulatory effect by DA was also observed in layer 5 pyramidal cells of the PFC tested in 30–50 days old mice (Ruan et al., 2014). Previous work also reported some differences between data obtained in rodents and in human cortex. For example, as already discussed above, Verhoog et al. (2013) observed only t-LTP in rodents for both negative and positive pre- and post-synaptic timing intervals, whereas pre before-post spike pairings evoke t-LTD and post-before-pre pairings elicit t-LTP at human cortical synapses. Moreover HCN1-channel-related gene expression and function is more prominent in human than mouse supragranular cortex (Kalmbach et al., 2018). This difference generates peculiar synaptic integration in human supragranular pyramidal neurons that could affect the effects of STDP.

Many presynaptic and/or postsynaptic factors could account for species-specific differences we observed. We have identified one of them, namely a differential impact onto inhibitory interneurons presynaptic to layer 5 pyramidal cells. Our data show that STDP induction alone potentiated IPSCs recorded from mature adult mice. We also observed that the peak amplitude of EPSPs overlapped with the initial part of the IPSP and it was sensitive to a GABA-A receptor antagonist. Therefore, this potentiation of synaptic inhibition could contribute to the t-LTD effect found in the EPSPs recorded in mature adult mice. Furthermore, application of DA during an STDP protocol depressed inhibitory transmission in the cortex of mature adult mice. This suggests that DA applied during an STDP protocol may exert at least some of its effect by disinhibition resulting in t-LTD of EPSP being converted to no change of synaptic strength in mature adult mice. This interpretation is consistent with our result that a D2R antagonist blocked the DA-mediated effect and with the previous result that D2Rs expressed by cortical inhibitory axonal terminals mediate the allosteric inhibition of GABA release and contribute to DA STDP gating in neocortical synapses of young adult mice (Xu and Yao, 2010). It is important to note, however, that the D2R-mediated modulation in young adult mice is effective at longer time interval (delta *T* = 30 ms), a time window that we have note tested in our study. In the human pyramidal neurons, STDP induction alone had no effect on IPSCs and we did not see a t-LTD effect in the EPSP such as the one seen in the mature adult mouse. Furthermore, when DA was applied during an STDP protocol, there was again no change in the IPSC, however we observed a potentiation in the EPSP, suggesting that presynaptic inhibition is not a target for DA modulation at human cortical circuits. Our data are consistent with data from the Allen Institute for Brain Science showing that DA receptors 1 and 2 have lower expression in human layer 5 cortical GABAergic neurons than in mouse.

Several other mechanisms may account for the species-specific differences we have observed. Amongst them, a key postsynaptic factor could be represented by the stronger compartmentalization of cortical layer 5 pyramidal neurons in humans than rodents (Beaulieu-Laroche et al., 2018), a feature that could be under neuromodulatory control. Ongoing and future work will provide more details on the specializations of cortical pyramidal neurons in humans as compared to rodents (Hodge et al., 2019; Berg et al., 2020; Gidon et al., 2020). They could lead to identification of differences in the integration of back-propagating APs and synaptic inputs resulting in human neurons specific STDP phenotypes.

Finally, it is important to acknowledge some methodological limitations present in our study. First, the extracellular stimulation method used precluded any defined information on the microcircuit investigated. During the experiments, the stimulation electrode was placed at layer 2–3, about 100–150 μm more superficial than the recording electrode placed in layer 5. Therefore, the stimulation electrode activated a heterogeneous set of extrinsic excitatory fibers (e.g., interhemispheric corticocortical afferents) and intrinsic axons (e.g., collaterals of pyramidal neurons). Moreover, the interneuron types directly or indirectly activated by the stimulation remained undefined (Kepecs and Fishell, 2014). Second, it is important to admit that the use of tissue from human cerebral cortex of patients subjected to neurosurgery has some intrinsic methodological limitations. One of these is that the tissue may have some pathological features that remain undetected. We have used cortical tissue from patients with low grade glioma tumor and although we recorded only from neurons located outside the main tumor, some infiltration cannot be excluded. Another possible limitation is the variability due to heterogeneity of cortical areas of provenance, different age and sex of the patients, their individual clinical and pharmacological history (see Table 2). Despite this variability, we observed basal functional parameters that were rather homogeneous across samples and patients and homogeneity in the effects mediated by DA, consistent with other reports testing pharmacological agents in human cortical slices (Komlósi et al., 2012; Bocchio et al., 2019; Kroon et al., 2019). Third, we recorded from layer 5 pyramidal neurons and pooled the data as these neurons were an homogeneous group. Recent data clearly indicate a pyramidal neuron type diversity based on genetic profile expression and projection sites (Hodge et al., 2019), but we did not attempt to take this aspect into account, as this would have been beyond the scope of our study. Fourth, DA gating has been associated with reward and prediction errors as well as novelty and salience detection (Palacios-Filardo and Mellor, 2019). We assume that the STDP model represents a cellular correlate underlying cognition, but how good is this assumption? One of the key unsolved issues is represented by the so-called distal reward problem, that is how neuronal networks, despite a temporal gap, identify which past networks activities led to reward and which are irrelevant (Izhikevich, 2007). One way to help solving this problem would be the identification of an eligibility trace generated by the STDP spiking activity and triggered by DA (Sutton and Barto, 1998), but this hypothesis awaits experimental demonstration. This seems a likely scenario since experimental evidence for plasticity eligibility traces has been already observed for the plasticity modulation induced by the monoamine receptors for norepinephrine and serotonin in the visual cortex (He et al., 2015).

**TABLE 2:** Donor patients attributes.

	Age	Sex	Histological diagnosis, tumor grade	Tumor IDH	Tumor localization, lateralization	Epilepsy, type	Anaesthesia
**1**	56–60	Male	Glioblastoma, 4	Wild type	Parietal, right	Yes, focal	Dexamethasone, Metaoxedrine/Phenylephrine, Prednisolone, Propofol, Remifentanil
**2**	76–80	Male	Glioblastoma, 4	Wild type	Temporal, right	No	Dexamethasone, Ephedrine, Fentanyl, Metaoxedrine/Phenylephrine, Propofol, Remifentanil
**3**	70–75	Female	Tissue necrosis	N/A	Temporal, right	No	Dexamethasone,Metaoxedrine/Phenylephrine, Propofol, Remifentanil
**4**	50–55	Male	Glioblastoma, 4	Wild type	Temporal, right	Yes focal	Dexamethasone,Metaoxedrine/Phenylephrine, Propofol, Remifentanil
**5**	60–65	Male	Glioblastoma, 4	Wild type	Temporal, left	No	Ephedrine, Metaoxedrine/Phenylephrine, Noradrenalin, Prednisolone, Propofol, Remifentanil
**6**	60–65	Female	Glioblastoma, 4	Wild type	Occipital, right	Yes, generalized	Dexamethasone, Ephedrine, Fentanyl, Metaoxedrine/Phenylephrine, Noradrenalin, Propofol, Remifentanil
**7**	70–75	Male	Glioblastoma, 4	Wild type	Temporal, right	No	Ephedrine, Metaoxedrine/Phenylephrine, Prednisolone, Propofol, Remifentanil
**8**	70–75	Female	Glioblastoma, 4	Wild type	Frontal, left	No	Dexamethasone, Metaoxedrine/Phenylephrine, Ephedrine, Fentanyl, Propofol, Remifentanil
**9**	60–65	Female	Glioblastoma, 4	Wild type	Parietal, left	No	Dexamethasone, Metaoxedrine/Phenylephrine, Propofol, Remifentanil
**10**	40–45	Male	Glioblastoma, 4	Wild type	Parietal, left	No	Alfentanil, Dexamethasone, Metaoxedrine/Phenylephrine, Fentanyl, Propofol, Remifentanil, Propofol
**11**	56–60	Male	Glioblastoma, 4	Wild type	Temporal, left	Yes, generalized	Alfentanil, Dexamethasone, Metaoxedrine/Phenylephrine, Ephedrine, Fentanyl, Propofol, Remifentanil
**12**	56–60	Male	Glioblastoma, 4	Wild type	Temporal, left	Yes focal	Ephedrine, Fentanyl, Metaoxedrine/Phenylephrine, Propofol, Remifentanil,
**13**	60–65	Male	Glioblastoma, 4	Wild type	Temporal, right	No	Dexamethasone, Ephedrine, Metaoxedrine/Phenylephrine, Prednisolone, Propofol, Remifentanil
**14**	70–75	Female	Glioblastoma, 4	Wild type	Temporal, right	No	Dexamethasone, Ephedrine, Fentanyl, Metaoxedrine/Phenylephrine, Propofol, Remifentanil
**15**	55–60	Male	Glioblastoma, 4	Wild type	Temporal, right	No	Dexamethasone, Ephedrine, Fentanyl, Metaoxedrine/Phenylephrine, Propofol, Remifentanil
**16**	80–85	Male	Glioblastoma, 4	Wild type	Temporal, right	No	Cefruroxim, Dexamethasone, Ephedrine, Fentanyl, Metaoxedrine/Phenylephrine, Propofol, Remifentanil
**17**	65–70	Female	Glioblastoma, 4	Wild type	Temporal, right	No	Cefruroxim, Metaoxedrine/Phenylephrine, Propofol, Remifentanil
**18**	75–80	Female	Gliobastoma, 4	Wild type	Parietal, right	No	Cefruroxim, Ephedrine, Fentanyl, Metaoxedrine/Phenylephrine, Propofol, Remifentanil

Despite these limitations, our findings, and particularly the discovery of DA modulation of STDP at excitatory synapses of layer 5 neurons in human cortex, provide information that can be further explored by future experiments. For example, the cellular correlates of DA-mediated gating of cognitive process in the human cortex could be explored by using an *in situ* multi-electrode array recording approach.

## Data Availability Statement

The original contributions presented in the study are included in the article/Supplementary Material, further inquiries can be directed to the corresponding author/s.

## Ethics Statement

The studies involving human participants were reviewed and approved by the Research Ethics Committee for the region of Middle Jutland (Denmark; project number M-2017-82-17). The patients/participants provided their written informed consent to participate in this study. The animal study was reviewed and approved by the Danish Animal Ethics Committee (2017-15-0201-01201).

## Author Contributions

EL: conceptualization, investigation, analysis, and writing. RJ: neurosurgery and commenting text. AK: neurosurgery and application to use human patients sample for research. JS: neurosurgery supervision, funding acquisition, and commenting text. MC: conceptualization, writing, funding acquisition, and supervision. All authors contributed to the article and approved the submitted version.

## Conflict of Interest

The authors declare that the research was conducted in the absence of any commercial or financial relationships that could be construed as a potential conflict of interest.

## Footnotes

1 http://www.nc3rs.org.uk/page.asp?id=1357
