# Supplementary material for: Dopaminergic Neuromodulation of Spike Timing Dependent Plasticity in Mature Adult Rodent and Human Cortical Neurons
Source: Front Cell Neurosci. 2021 Apr 22;15:668980. doi: 10.3389/fncel.2021.668980 (PMC8102156; doi:10.3389/fncel.2021.668980)
Supplement: Supplementary file 1 [file Data_Sheet_1.pdf]

Dopaminergic neuromodulation of spike timing dependent plasticity in mature adult rodent and human cortical neurons

Emma Louise Louth[1], Rasmus Langelund Jørgensen[2], Anders Rosendal Korshoej [2], Jens Christian Hedemann Sørensen[2] and Marco Capogna[1*]

[1]Department of Biomedicine, DANDRITE and PROMEMO, Aarhus University, Denmark; [2] Department of Neurosurgery, Aarhus University Hospital, Denmark

Supplementary Files

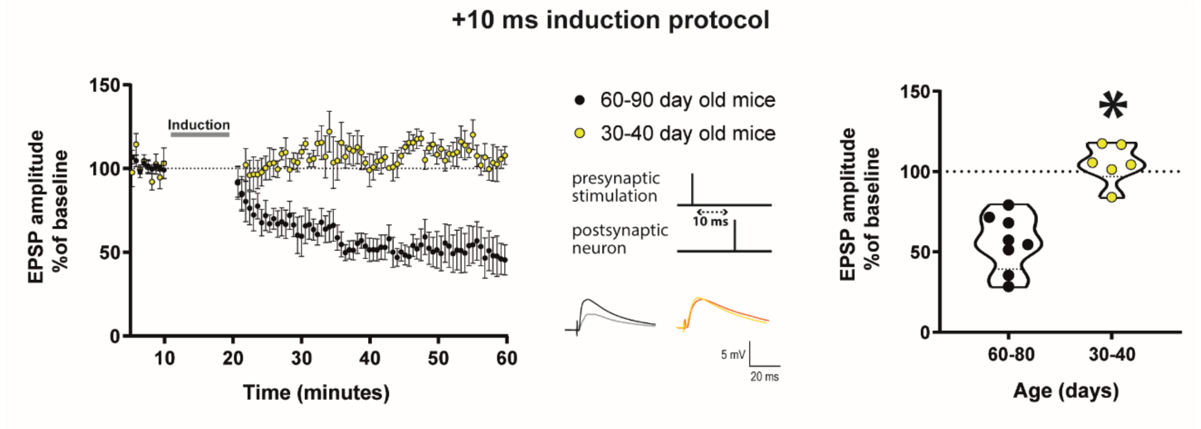

Figure Supplement 1 (Supplement to Fig 1)

The peak amplitude of EPSPs following at $\Delta\tau$ +10ms STDP induction protocol in mature adult and adult mouse layer 5 cortical pyramidal neurons. Left, the time-course of the EPSP peak amplitude during the STDP experiment in both groups of mice. Middle, the STDP induction protocol timing is illustrated and below are example traces of EPSPs. The baseline trace is the darker trace, the trace following STDP induction is the lighter trace; each trace is the average of 80 traces from the same recording. Right, violin plots of summary data showing that mature adult mice exhibited t-LTD (53.4 ± 8.1, p = 0.02 vs 100 %) whereas adult mice exhibited no change (104.9 ± 5.0, p = 0.2 vs 100 %). There was a significant difference between EPSP peak amplitude following STDP induction in mature adult and adult mice (p = 0.004). All data are shown as mean ± SEM. Number of neurons recorded in mature adult mice, n= 8, and adult mice n= 6.

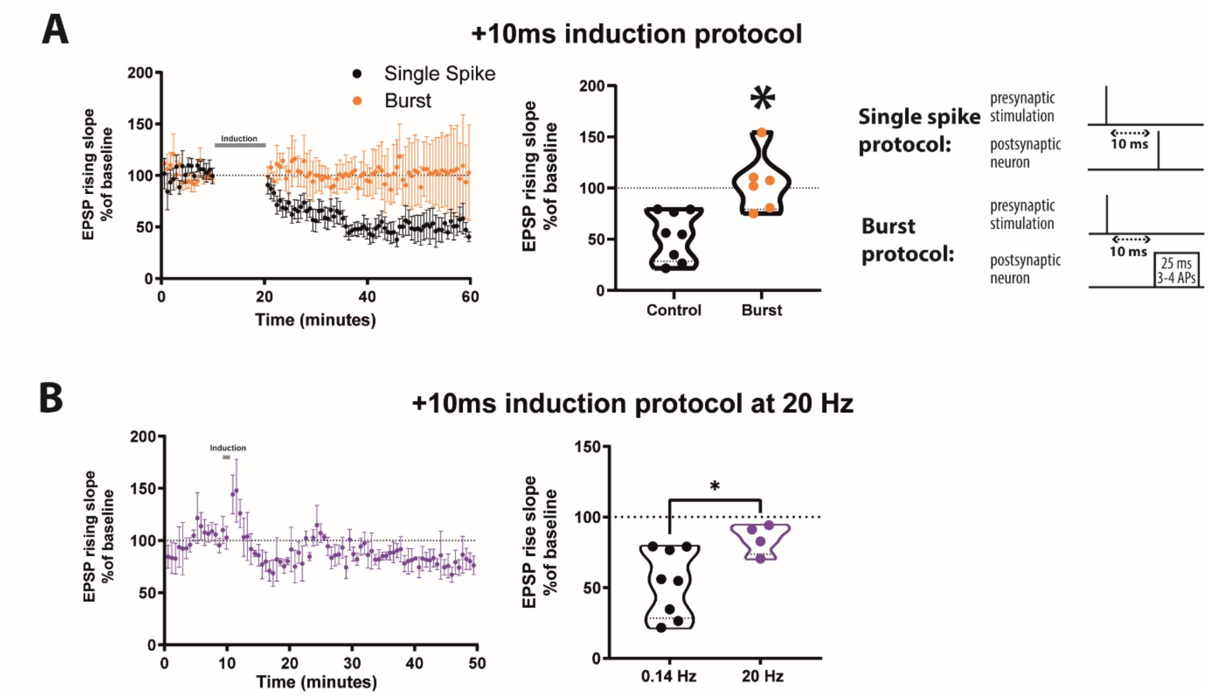

Figure Supplement 2 (Supplement to Fig 1)

A burst of postsynaptic APs STDP induction protocol blocks t-LTD in mature adult mice. **(A)** The time-course of the EPSP rising slope during the STDP experiment is shown to the left. Violin plots of summary of the data showing a significant difference between EPSP rising slope using a single spike or burst protocol (*p* = 0.003) are shown in the middle. The STDP induction protocol timing is illustrated to the right (B) Induction by stimulating at 20 Hz results in no change in EPSP rise slope (84.7 ± 5.3, p = 0.1 vs 100 %) and is significantly different than the control 0.14 Hz protocol used in the rest of the study (p = 0.03). The time-course of the EPSP rising slope during the STDP experiment is shown to the left. Violin plots of summary of the data are shown to the right All data are shown as mean ± SEM. For the single spike protocol n = 8, for the burst protocol n = 6 and for the 20 Hz protocol n = 4.

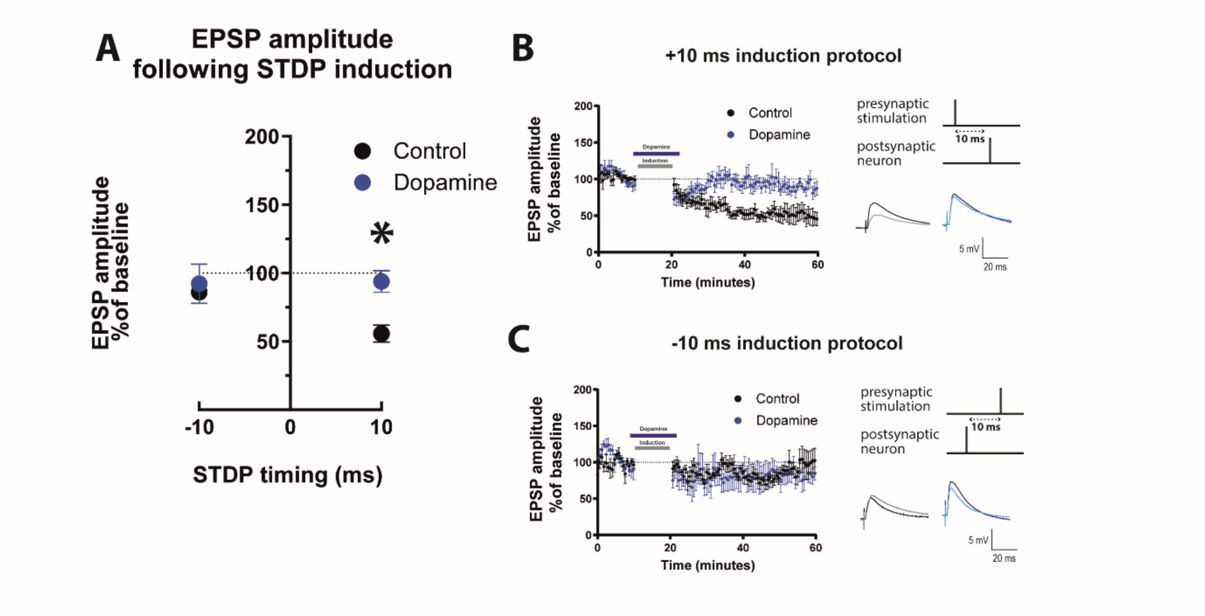

Figure Supplement 3 (Supplement to Fig 2)
Effect of DA on EPSP amplitude following STDP induction in mature adult mouse cortical layer 5 pyramidal neurons. **(A)** STDP induction at $\Delta\tau$ -10 ms and + 10 ms EPSP-AP pairing timings with and without 20 µM DA application. EPSP amplitude remained unchanged with the -10ms timing for both the control (88.5 ± 4.7, *p* = 0.6 vs 100%) and DA groups (92.2 ± 14.3, *p* = 0.6 vs 100%) and they were not significantly different from each other (*p* = 0.8). At the +10 ms timing, EPSP amplitude was significantly different between the groups (*p* = 0.005), resulting in t-LTD in the control group (55.7 ± 6.2, p = 0.008 vs 100%) and no change in the DA group (93.8 ± 7.9, *p* = 0.6 vs 100 %). The time-course of the EPSP amplitude during the STDP experiment using the +10 ms timing is shown in **(B)** and the -10 ms timing in **(C)**. DA bath application and time of STDP induction are indicated by bars in the graph. To the right, the STDP induction protocol timing is illustrated and below are example traces of EPSPs. The baseline trace is the darker trace, the resultant trace following STDP induction is the lighter trace; each trace is the average of 80 traces from the same recording. All data are shown as mean ± SEM. For the -10ms timing n = 6 and for the +10ms timing n=8.

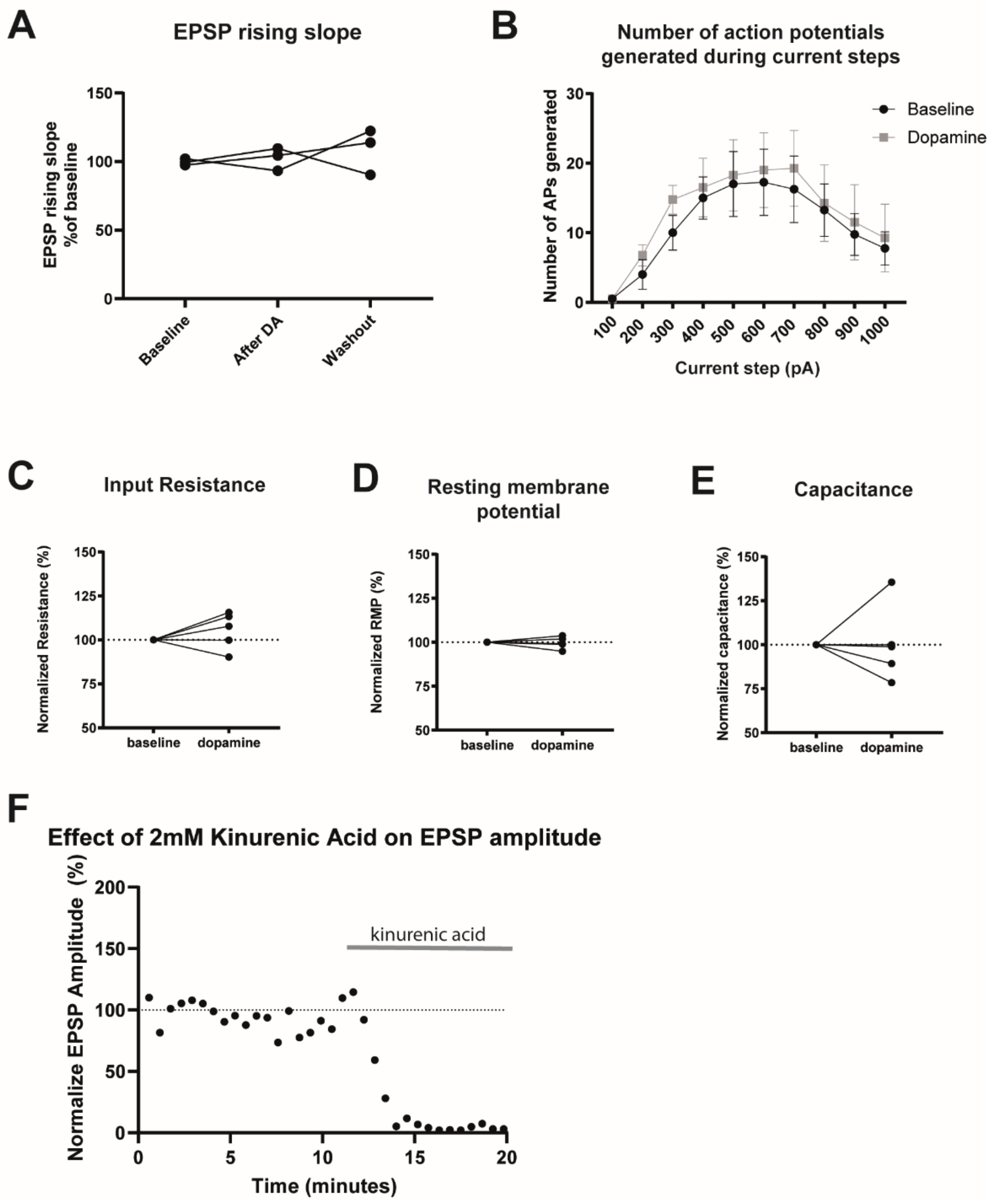

Figure Supplement 4 (Supplement to Fig 2)

DA does not affect low frequency-evoked EPSP or basic electrophysiological properties. **(A)** While evoking EPSPs at 0.14 Hz, the same rate as baseline stimulation before STDP induction, 20 µM DA was bath applied without STDP induction for seven minutes. Data points were taken one minute before DA application (control), during the final minute of DA application (DA data) and ten minutes after the end of DA application (washout). DA application had no effect on EPSP rising slope (Friedman test, $p = 0.9$). We also measured effect of DA on AP firing frequency (B, two-way ANOVA, effect of DA: $p = 0.3$ $F_{(1, 60)} = 1.2$, effect of current step: $p = 0.0002$ $F_{(9, 60)} = 4.5$, interaction: $p = 0.9$ $F_{(9, 60)} = 0.05$), input resistance (C, DA: 105.4 ± 4.6, $p = 0.4$ vs 100%), resting membrane potential (D, DA: 99.6 ± 1.5, $p = 0.9$ vs 100%) and capacitance (E, DA: 100.4 ± 9.6, $p = 0.9$ vs 100%). DA did not significantly affect these parameters. **(F)** Application of 2mM kinurenic acid completely abolished the EPSP. Individual data points are shown, except in B where data is shown as mean ± SEM. For EPSP rising slope, n = 3. For the remaining n = 5.

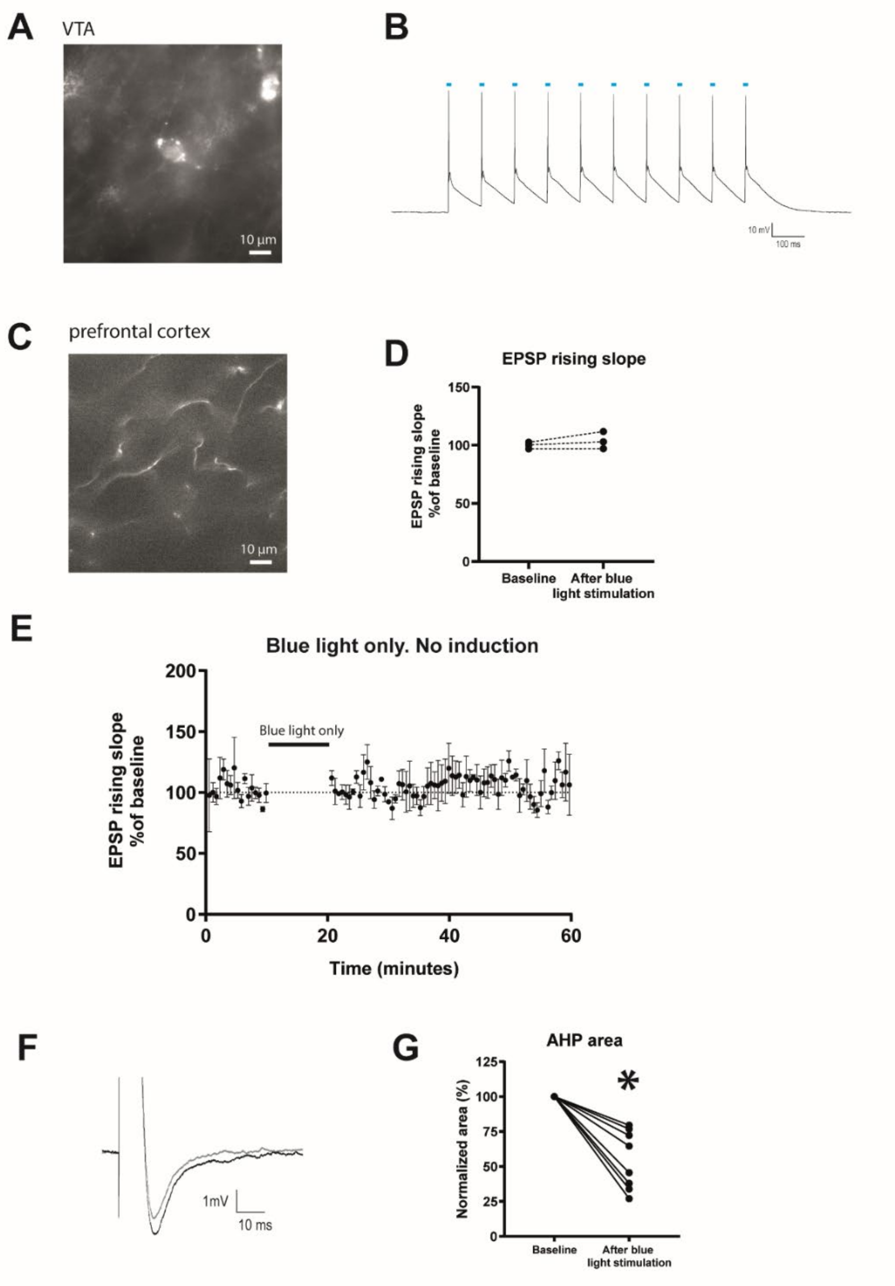

Figure Supplement 5 (Supplement to Fig 3)
Viral transfection of ChR2 in dopaminergic neurons of the VTA from Dat^{IREScre} mice. **(A)** An example of a VTA cell with viral expression as demonstrated by eYFP fluorescence. **(B)** Optogenetic stimulation of these cells with a 10 Hz pulse train of blue light pulses (460 nm, ~10mW power, shown as blue bars) evoked APs in current clamp mode. **(C)** example of prefrontal cortex fibers from VTA neurons as demonstrated by eYFP fluorescence. **(D)** Blue light stimulation alone had no effect on EPSP rising slope ($p = 0.3$, n = 3). **(E)** The time course of the blue light stimulation only is shown. **(F)** Averaged AHP traces before and after blue light stimulation. The baseline trace is the darker trace, the after trace is the lighter trace; each trace is the average of 3 traces from the same recording. **(G)** AHP area analysis showing that AHP area is decreased following blue light stimulation ($p = 0.0008$, n = 8).

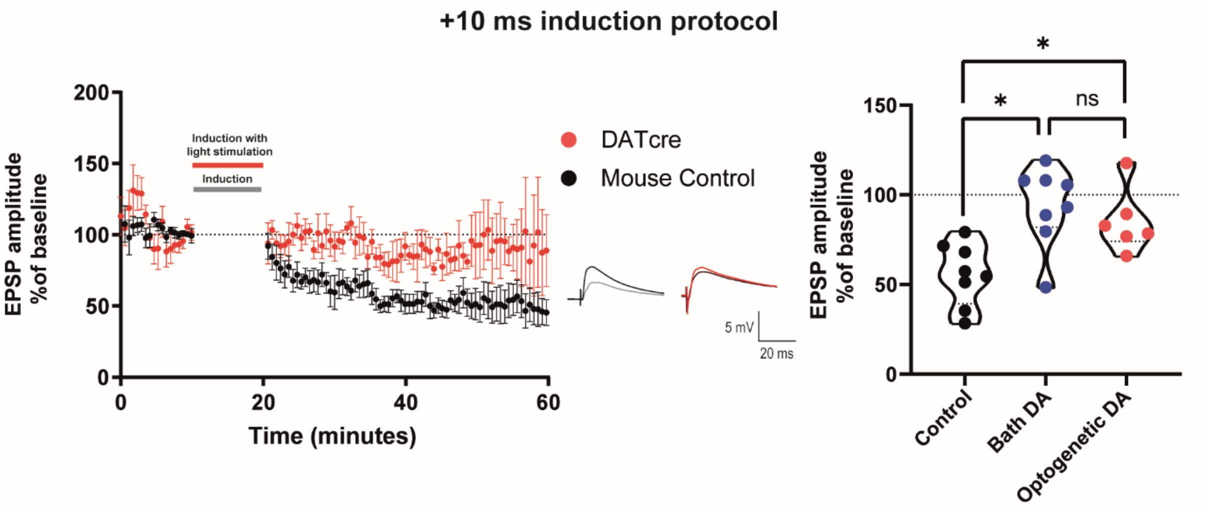

Figure Supplement 6 (Supplement to Fig 3)
Optogenetically triggered DA release during STDP induction blocks EPSP peak amplitude t-LTD in cortical layer 5 pyramidal neurons of mature adult mouse. The time-course of the EPSP peak amplitude during the STDP experiment in both control and Dat^{IREScre} mice (left) and violin plots of summary of the data (right). Example EPSP traces are shown (middle). The baseline trace is the darker trace, the after trace is the lighter trace; each trace is the average of 80 traces from the same recording. Data show that, similar to bath application of DA, optogenetically triggered release of DA shows no change in EPSP amplitude (85.2 ± 7.2, $p = 0.2$ vs 100 %,). A significant difference between the control group and the DA exposed groups was detected (Kruskal-Wallis test, $p = 0.003$, Dunn's multiple comparison test, control vs bath application of dopamine: $p = 0.006$, control vs optogenetic DA: $p = 0.04$, bath vs optogenetic application of DA: $p = 0.9$). All data are shown as mean ± SEM. For the optogenetic stimulation n = 6, while for the control and bath DA groups n = 8.

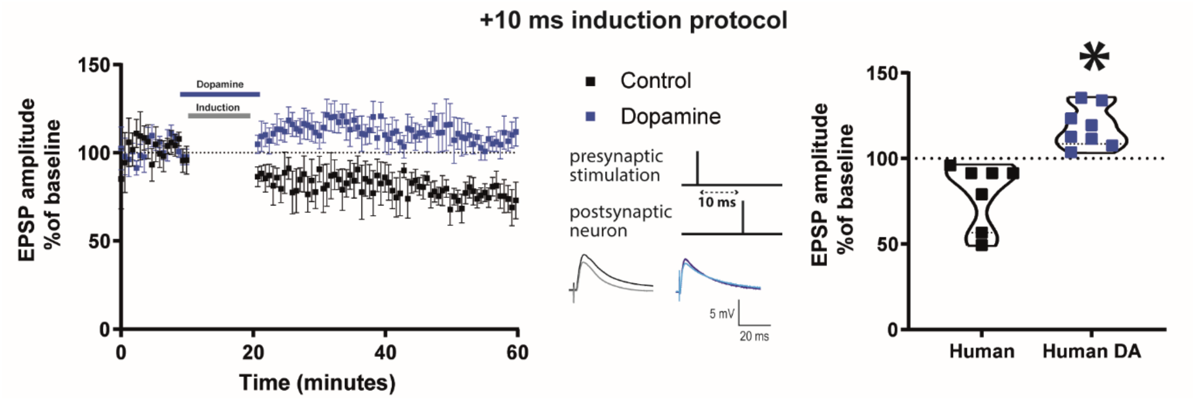

Figure Supplement 7 (Supplement to Fig 4)

DA potentiates baseline EPSP peak amplitude after Δτ +10ms STDP protocol in adult human cortical layer 5 pyramidal neurons. Left, the time-course of the EPSP amplitude after Δτ +10ms STDP induction protocol. Middle, the STDP induction protocol timing is illustrated and below are example traces of EPSPs. The baseline trace is the darker trace, the resultant trace following STDP induction is the lighter trace; each trace is the average of 80 traces from the same recording. Right, violin plots of summary of the results showing t-LTD in the control group (79.4 ± 7.1, $p = 0.02$ vs 100 %) and t-LTP in the DA group (118.5 ± 4.2, $p = 0.008$ vs 100 %). There was a significant difference between EPSP amplitude with and without DA application during STDP induction ($p = 0.0003$). All data are shown as mean ± SEM. For the control group n=7 and for the DA group n = 8.

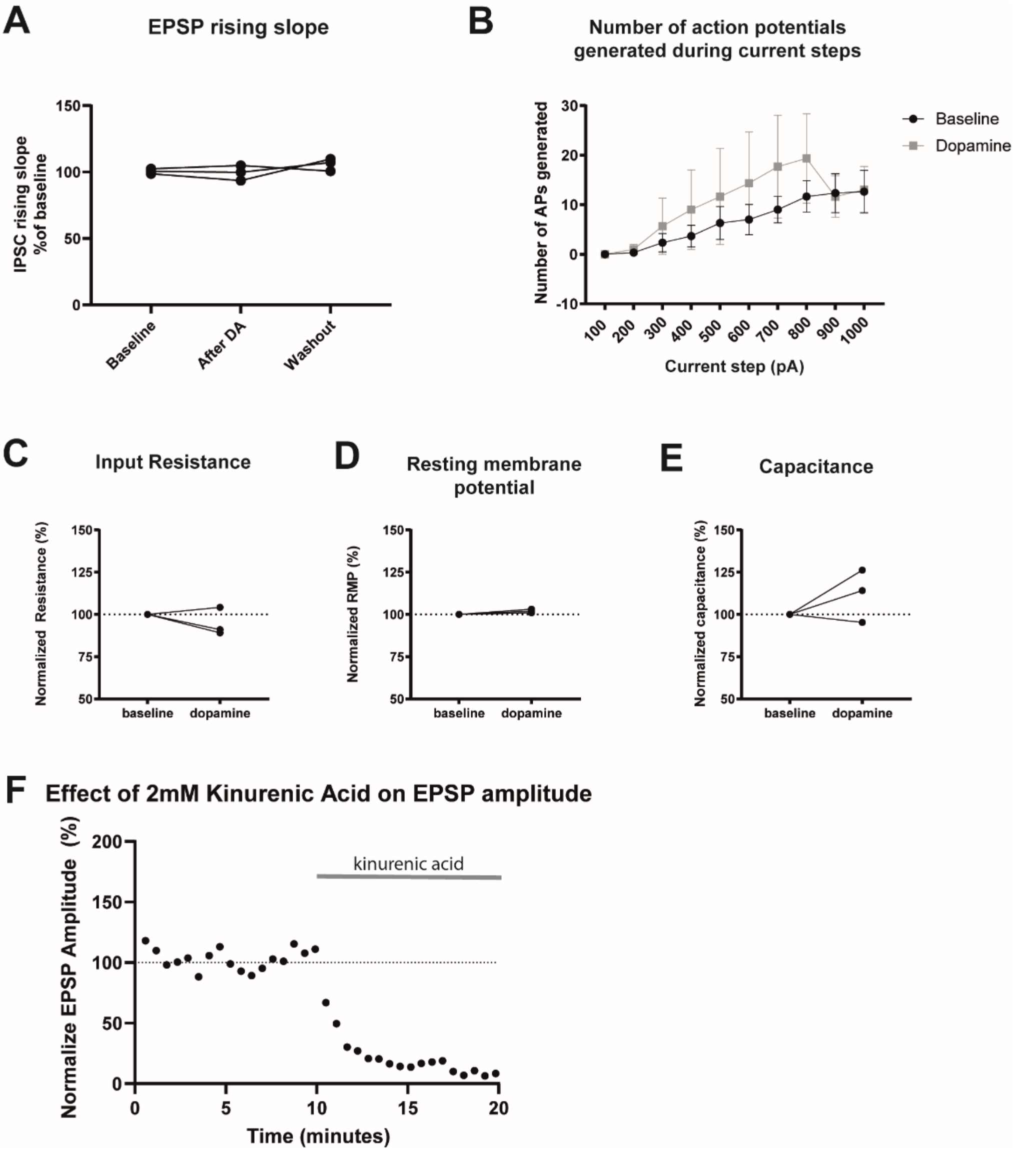

Figure Supplement 8 (Supplement to Fig 4)

DA does not affect low frequency-evoked EPSP or basic electrophysiological properties recorded from layer 5 pyramidal neurons of human cortex. **(A)** EPSPs were evoked at 0.14 Hz, the same rate as the baseline protocol; DA was bath applied without STDP induction for seven minutes. Data points were taken one minute before DA application (control), during the final minute of DA application (DA data) and ten minutes after the end of DA application (washout). DA had no effect on EPSP rising slope (Friedman test, $p = 0.9$). We also measured the action of DA on AP firing frequency (B, two-way ANOVA, effect of dopamine: $p = 0.1$ $F_{(1, 40)} = 2.4$, effect of current step: $p = 0.07$ $F_{(9, 40)} = 2.0$, interaction: $p = 0.9$ $F_{(9, 40)} = 0.2$), input resistance (C, dopamine: 94.8 ± 4.7, $p = 0.5$ vs 100%), resting membrane potential (D, dopamine: 101.9 ± 0.7, $p = 0.3$ vs 100%) and capacitance (E, dopamine: 111.9 ± 9.0, $p = 0.5$ vs 100%). DA did not significantly affect these parameters. **(F)** Application of 2mM kinurenic acid completely abolished the EPSP. Individual data points are shown, except in B where data is shown as mean ± SEM. For all groups, n=3.

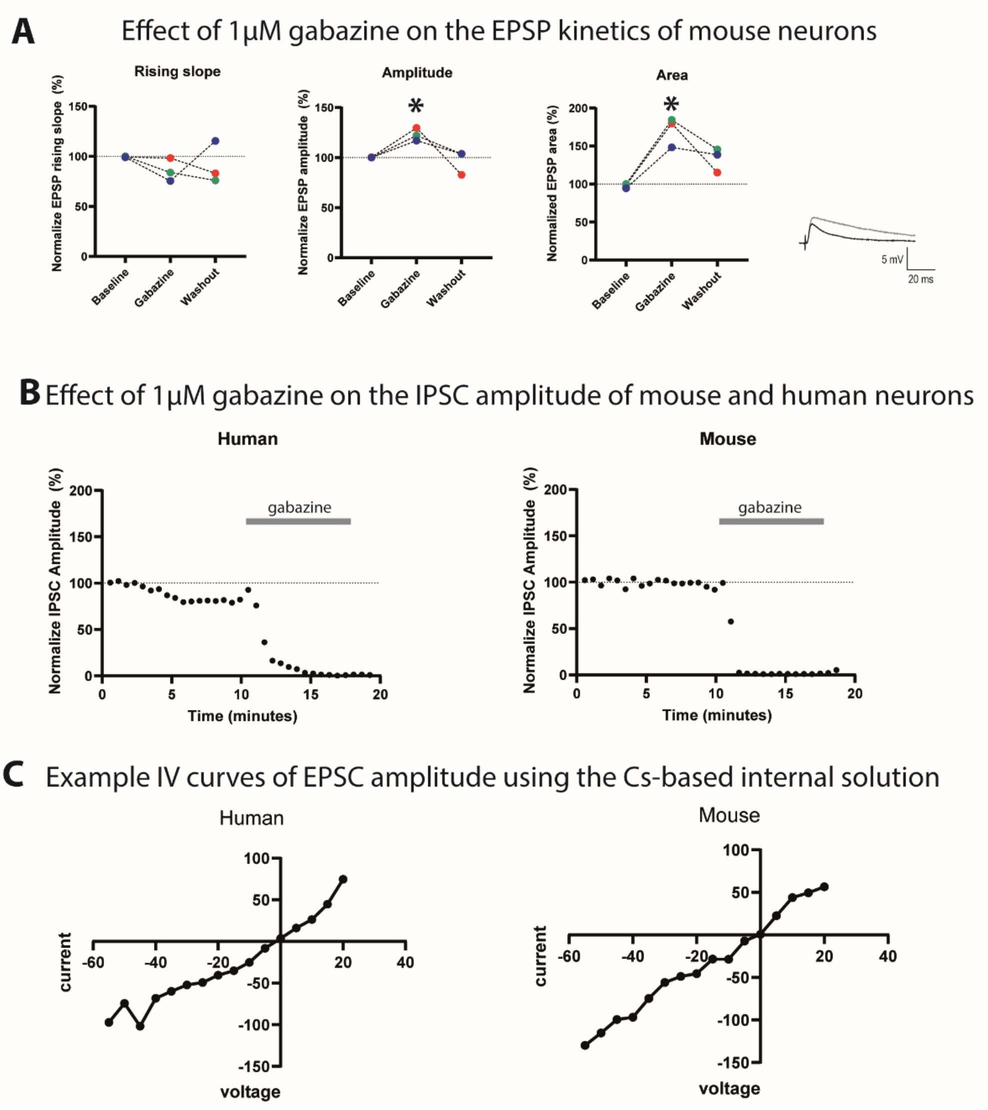

Figure Supplement 9 (Supplement to Fig 5)
The GABA-A receptor antagonist gabazine (1 µM) affects EPSCs and abolished IPSCs in human and mouse cortical layer 5 pyramidal neurons. **(A)** Gabazine application, showed a trend to increased EPSP peak amplitude (middle, Friedman test, p = 0.2Dunn's multiple comparison baseline vs gabazine p = 0.08, baseline vs washout p = 0.9) and enhanced area (right, Friedman test, p = 0.03, Dunn's multiple comparison baseline vs gabazine p = 0.03, baseline vs washout p = 0.4), and did not affect rising slope (left, Friedman test, p = 0.5, , Dunn's multiple comparison baseline vs gabazine p = 0.4, baseline vs washout p = 0.4). Individual data points are shown (n = 3), example traces are shown on the far right. The baseline trace is the darker trace, the resultant trace following gabazine application is the lighter trace; each trace is the average of five traces. **(B)** Recorded IPSCs are abolished by gabazine application (denoted by grey bars). Individual example traces in human (left) and mouse (right) are shown. **(C)** IV curves of EPSC amplitude using the Cs-based internal solution. Individual example traces in human (left) and mouse (right) are shown.

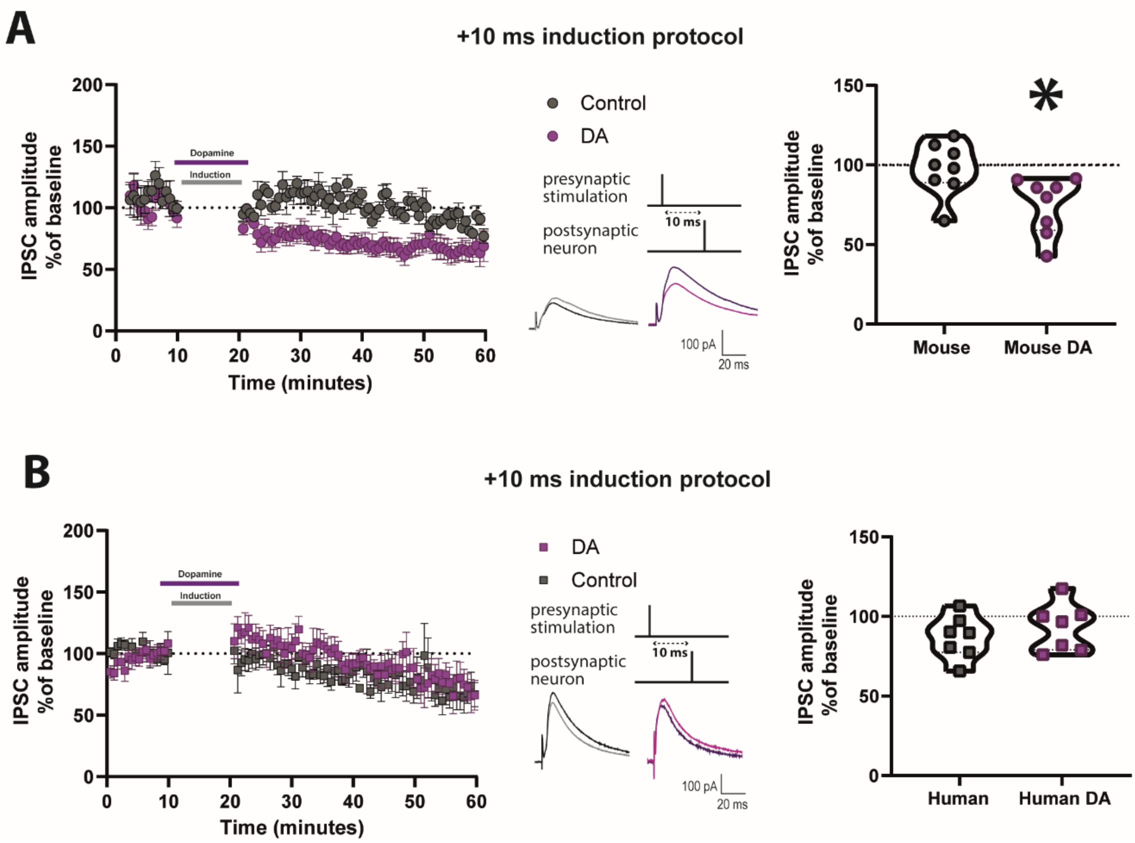

Figure Supplement 10 (Supplement to Fig 5)

DA reduces IPSC amplitude after Δτ +10ms STDP protocol in cortical layer 5 pyramidal neurons of mature adult mice but not humans. Effect of DA on IPSC amplitude following STDP induction in adult mouse (A; control: 97.5 ± 5.9, p = 0.9 vs 100%; DA: 74.7 ± 6.3, p = 0.008 vs 100%) and human (B; control: 87.2 ± 5.3, p = 0.08 vs 100%; DA: 93.1 ± 5.7, p = 0.3 vs 100%) pyramidal neurons. Left, the time-course of the IPSC amplitude after Δτ +10ms STDP timing protocol. Middle, the STDP induction protocol timing is illustrated and below are example traces of IPSCs. The baseline trace is the darker trace, the resultant trace following STDP induction is the lighter trace; each trace is the average of 80 traces from the same recording. Right, violin plots showing summary of the results. DA significantly reduced the IPSC amplitude after STDP induction in neurons recorded from mature adult mice (p = 0.01, n = 8), but not from humans (p = 0.6, n = 7). All data are shown as mean ± SEM.

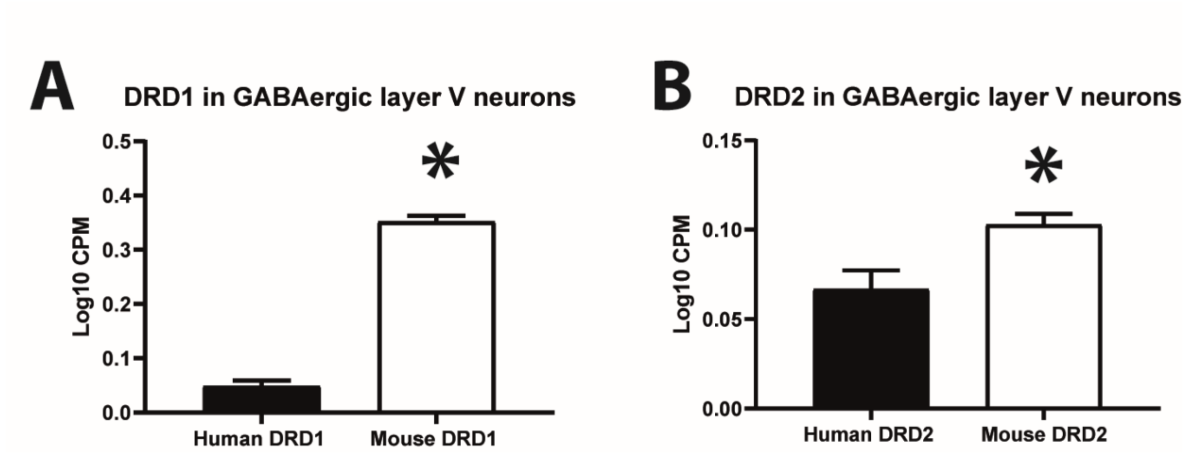

Figure Supplement 11 (Supplement to Fig 5)
DA receptor expression data in cortical layer 5 neurons from the Allen Brain Atlas of RNA-seq data. **(A)** DRD1 expression in cortical layer 5 GABAergic neurons from human (0.05 ± 0.01, n = 786) and mouse (0.4 ± 0.01, n = 3504) are significantly different (p = 0.0001). **(B)** DRD2 expression in cortical layer 5 GABAergic neurons from human (0.07 ± 0.01, n = 786) and mouse (0.1 ± 0.006, n = 3504) are significantly different (p = 0.003). Data were not normally distributed and therefore analyzed with Welch's t test.

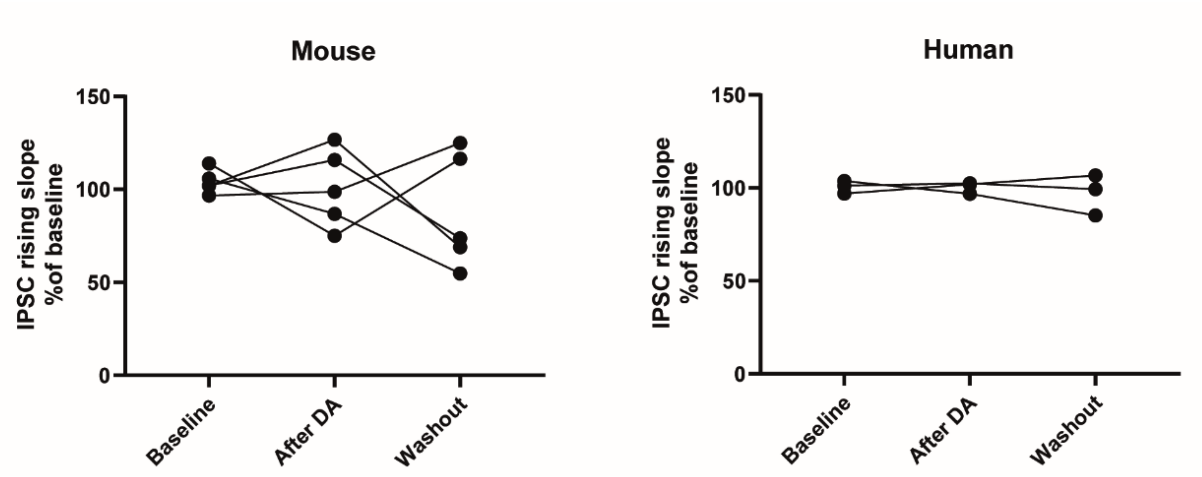

Figure Supplement 12 (Supplement to Fig 5)
Action of DA on IPSC rising slope in mouse and human pyramidal neurons. DA was applied for seven minutes while evoking IPSCs at 0.14 Hz, that was the same repetition rate used to collect baseline IPSCs before the STDP protocol. Data points were taken one minute before DA application (control), during the final minute of DA application (DA data) and ten minutes after the end of DA application (washout). DA had no effect on IPSC rising slope in mouse (Friedman test, p = 0.9, , n = 5) and human (Friedman test, p = 0.9,n = 3) neurons. Individual data points are shown.

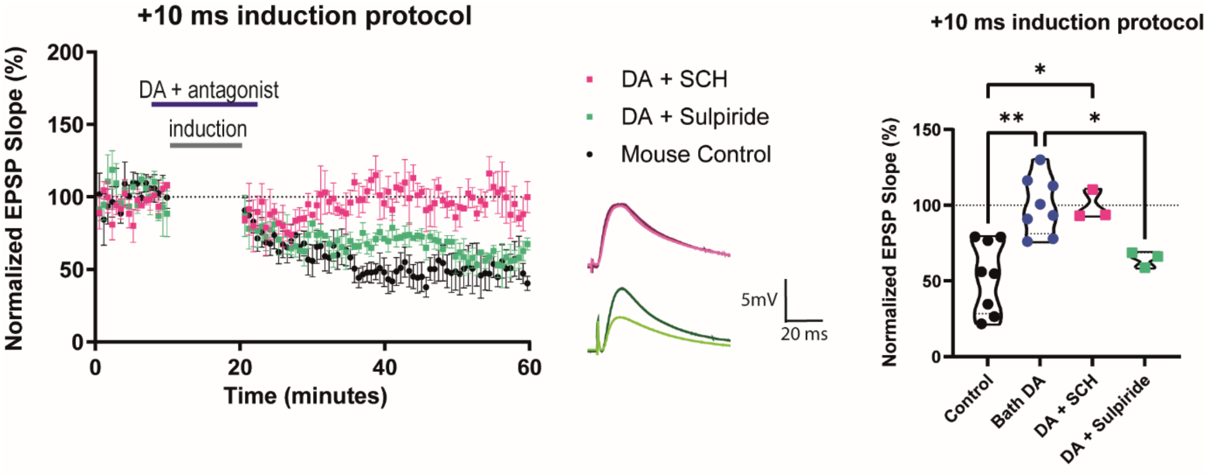

Figure Supplement 13 (Supplement to Fig 5)
The D2R antagonist sulpiride prevents DA potentiation of STDP at $\Delta\tau$ = +10ms STDP protocol in mouse cortical layer 5 pyramidal neurons. Left, the time-course of the EPSP slope after $\Delta\tau$ = +10ms STDP induction protocol. Middle, example traces of EPSPs are shown. The baseline trace is the darker trace, the resultant trace following STDP induction is the lighter trace; each trace is the average of 80 traces from the same recording. Right, violin plots of summary of the results showing significant effects by Kruskal-Wallis test (p = 0.004). By Dunn's multiple comparisons test the control group was significantly different from the D1 antagonist SCH group (p = 0.01) but not from the sulpiride group (p = 0.9). Bath application of DA alone was significantly different from the sulpiride group (p = 0.04) but no the SCH group (p = 0.8). The sulpiride and SCH groups showed a trend but were not significantly different from each other (p = 0.05). All data are shown as mean ± SEM.
